# Liquid biopsy in breast cancer: Redefining precision medicine

**DOI:** 10.1016/j.jlb.2025.100312

**Published:** 2025-07-16

**Authors:** Maria Luisa Schiavone, Rosa Scarpitta, Francesco Ravera, Sara Bleve, Carolina Reduzzi, Federico Di Cocco, Martina Dameri, Gabriele Zoppoli, Antonio Giuseppe Naccarato, Pier Vitale Nuzzo, Massimo Cristofanilli, Giuseppe Nicolò Fanelli, Cristian Scatena

**Affiliations:** a Division of Pathology, Department of Translational Research and New Technologies in Medicine and Surgery, University of Pisa, 56126, Pisa, Italy; b Department of Internal Medicine and Medical Specialties, University of Genova, 16132, Genova, Italy; c Department of Pathology and Laboratory Medicine, New York Presbyterian Hospital, Weill Cornell Medicine, New York, NY, USA; d Department of Medical Oncology, IRCCS Istituto Romagnolo per lo Studio dei Tumori “Dino Amadori”, Meldola, Italy; e Department of Medicine, Weill Cornell Medicine, Englander Institute for Precision Medicine, New York Presbyterian Hospital, New York, NY, 10021, USA; f Ospedale Policlinico San Martino, IRCCS, 16132, Genova, Italy; g Department of Oncology, Pisa University Hospital, 56126, Pisa, Italy

**Keywords:** Breast cancer, Liquid biopsy, Circulating tumor cells, Circulating tumor DNA

## Abstract

Breast cancer (BC) is the most frequent cancer and the leading cause of cancer-related death among women worldwide. It represents a heterogeneous group of diseases with distinct morphological, immunophenotypic, and molecular profiles, which significantly impact clinical behavior and therapeutic response. Moreover, under treatment pressure, tumor cells may undergo molecular changes and phenotypic plasticity, leading to resistance and therapeutic failure. Although tissue biopsy remains the gold standard for diagnosis and molecular characterization, it has several limitations, including invasiveness, sampling bias, and the inability to dynamically capture tumor evolution over time. Hence, a non-invasive and repeatable approach capable of real-time monitoring is increasingly needed.

Liquid biopsy (LB), through the analysis of circulating tumor cells (CTCs) and circulating tumor DNA (ctDNA), has emerged as a powerful tool to complement tissue biopsy. It allows for longitudinal assessment of tumor burden, detection of minimal residual disease, and identification of molecular alterations relevant to targeted therapies. Despite promising results, the integration of LB into clinical practice is still limited by methodological heterogeneity, standardization gaps, and regulatory issues. Nonetheless, LB represents a key advancement toward precision oncology and may become essential in the personalized management of BC patients.

In this review, we explore the current applications, benefits, and technical limitations of LB in different BC settings. We provide a comprehensive overview of the biological and clinical significance of CTCs and ctDNA, emphasizing their diagnostic, prognostic, and predictive roles. Finally, we present an updated summary of ongoing clinical trials that incorporate LB for clinical decision-making.

## 1 Introduction

Breast cancer (BC) is the most common malignancy among women worldwide, with 2.3 million new cases and 685.000 cancer-related deaths globally [1]. The main causes of BC mortality are distant metastases, tumor relapse, and drug resistance [2]. According to the Surveillance, Epidemiology, and End Results (SEER) program (https://seer.cancer.gov/), the 5-year survival rate for localized BC patients is 99 %. However, approximately 20–30 % of primary BC patients relapse after treatment [3], and the 5-year survival rate falls to 28 % in patients who develop distant metastasis [4]. Therefore, identifying patients at high risk for distant metastasis is mandatory to improve clinical outcomes, and early detection remains one of the most effective strategies.

BC encompasses a heterogeneous group of malignancies with different morphological and immunophenotypic features and distinctive molecular landscapes. Thus, the epi/genomic, transcriptomic, and proteomic complexity affects clinical behavior and disease progression [5].

BC is currently routinely subclassified according to the expression of estrogen (ER) and progesterone (PgR) receptors, human epidermal growth factor receptor 2 (HER2), and proliferation index assessed directly on tissue specimens. These biomarkers have strong prognostic and predictive implications, highlighting the upregulated signaling pathways that can be targeted with medical therapies [6,7]. Despite treatment efforts, tumor cells can adapt phenotypically and molecularly under the selective pressure of therapy, ultimately resulting in treatment resistance and failure [8,9]. Therefore, defining the tumor's gene expression profile at different time points during treatment is an ever-growing need [10].

Over the past decade, cutting-edge technologies have shed light on the complex molecular basis of cancer heterogeneity and the mechanisms underlying metastatization [[11], [12], [13], [14]]. Among them, liquid biopsy (LB) has drastically revolutionized the field of clinical oncology, opening new horizons for improving clinical decision-making [[15], [16], [17], [18], [19]]. Since the discovery that invasive carcinomas release into the bloodstream proteins, tumor cells, fragments of DNA, RNA, and extracellular vesicles [20], a new generation of biomarkers has become available as an alternative tool for early cancer detection and treatment monitoring [21], [22], [289].

**Fig. 1:**
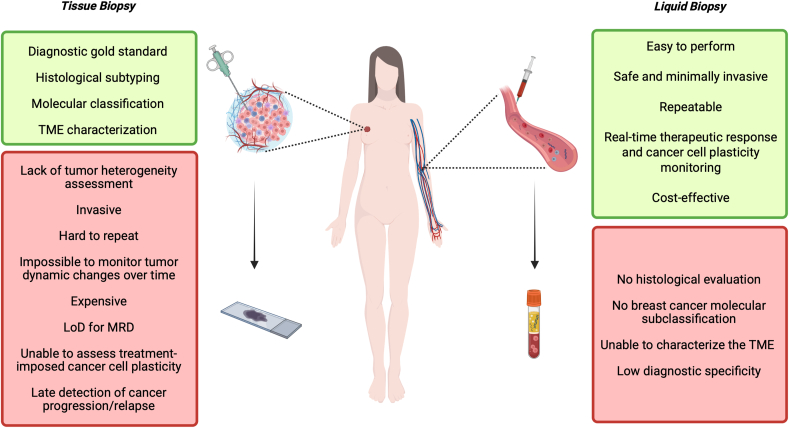
Schematic representation of the strengths and weaknesses of tissue and liquid biopsy approaches. TME: tumor microenvironment; LoD: Limit of Detection; MRD: Minimal Residual Disease. *Created with BioRender.com.*

Currently, the gold standard for BC diagnosis and its molecular characterization is still tissue biopsy; however, this approach has several limitations such as: (i) the characterization of the epi/genetic and phenotypic background of the whole tumor may be inaccurate [12,20,[23], [24], [25]]; (ii) tissue biopsy could be costly, painful, hard to repeat, and potentially risky for the patient; (iii) the clonal evolution phenomenon is hard to evaluate during tumor progression [26,27]. Therefore, it's clear that a single conventional tissue biopsy may not provide accurate evidence about the most appropriate therapy [28]. LB may overcome these limitations [29,30]. Fig. 1 summarizes the benefits and drawbacks of both approaches.

This review aims to assess and summarize the utility of LB in BC patients. Firstly, we will provide a detailed description of circulating biomarkers such as circulating tumor cells (CTCs) and circulating tumor DNA (ctDNA); then, we will recapitulate the diagnostic, prognostic, and predictive value of circulating biomarkers, including their advantages and limitations; finally, we will discuss ongoing trials that use CTCs or ctDNA assessment for patients' randomization, therapy management, and response evaluation.

## 2 Methodology

In this non-systematic literature review, we selected original articles, reviews, systematic reviews, metanalysis, and editorials, published in English language, with no year specification till February 2025. The most significant and recent papers were then chosen to gain insight into the current state-of-the-art, together with ongoing studies regarding CTCs and ctDNA as potential biomarkers for BC diagnosis, prognosis, and prediction.

Using ClinicalTrials.gov platform, we identified trials involving CTCs or ctDNA in BC patients. We decided to highlight trials in which patients’ randomization or allocation to therapy is secondary to CTCs or ctDNA assessment, or in which CTCs or ctDNA represent the outcome measure for assessing the response to treatment.

## 3 Liquid biopsy in breast cancer: an old concept to be renewed

**Fig. 2:**
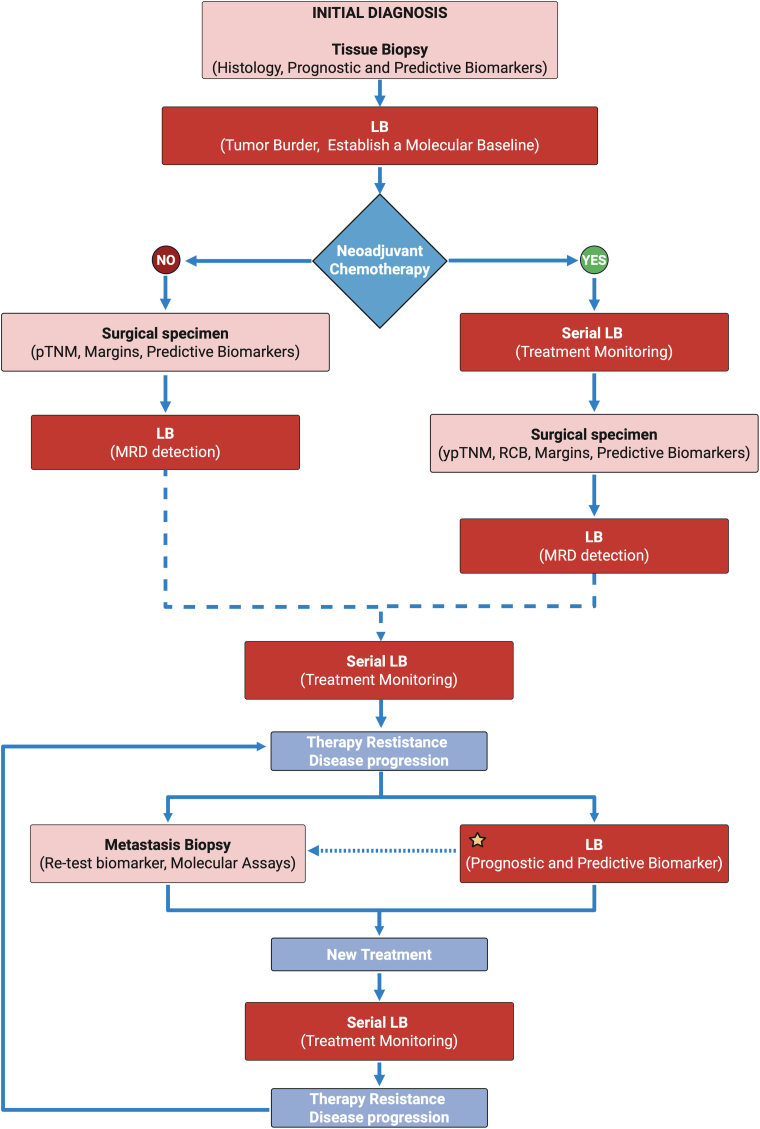
Integrated clinical workflow combining tissue and liquid biopsy (LB) in breast cancer patients' management. The algorithm illustrates the role of LB across different timepoints, including baseline profiling, treatment monitoring, and minimal residual disease detection. The star symbol indicates the only LB application currently approved in clinical practice. Dashed arrows represent time-dependent steps such as MRD surveillance or late treatment adaptation. The dotted arrow suggests that, in real-world settings, a reflex test on metastatic tissue biopsy may still be required in case of inconclusive or uninformative liquid biopsy results (e.g., low ctDNA levels or non-informative profiles). pTNM/ypTNM: pathological tumor staging (pre/post-chemotherapy); RCB: residual cancer burden; MRD: minimal residual disease. *Created with*BioRender.com.

LB is a promising approach with noteworthy potential for early detection and treatment monitoring in several malignancies [31]. LB approach is based on the isolation of entities derived from the tumor's site such as proteins, CTCs, DNA/RNA fragments, and extracellular vesicles, present in the body fluids of cancer patients [32]. Therefore, by leveraging the analysis of their genomic and proteomic data, tumor-derived components can provide crucial information on both primary (non-metastatic) and advanced cancers [33]. The main advantages of LB include its repetitive nature, minimal invasiveness, ease of execution, and cost-effectiveness [34]. However, nowadays LB remains an assay complementary to conventional tissue biopsy in early cancer detection but can provide tumor burden assessment and real-time monitoring. Based on this concept, we propose a clinically oriented algorithm (Fig. 2) that integrates tissue biopsy and liquid LB throughout the diagnostic–therapeutic journey of BC patients.

Since the increase of specific circulating tumor biomarkers in serial blood sampling has been proposed as an indicator of relapse [35], cancer antigens have been frequently used to monitor relapse after surgery in primary disease and response to therapy in advanced disease [36]. Among them, Food and Drug Administration (FDA) approved Cancer Antigen 15-3 (CA15-3) and Carcinoembryonic Antigen (CEA) as reliable biomarkers in BC follow-up [37].

CA 15-3 is a high-molecular-weight glycoprotein polymorphic epithelial mucin (PEM), also known as episialin, which is produced by the *MUC1* gene and plays a pivotal role in cell adhesion. In BC, PEM is often overexpressed and abnormally glycosylated and may contribute to promote metastasis. Moreover, increased levels of CA15-3 are more strongly related to shorter progression-free survival (PFS) and overall survival (OS) than tumor size and nodal status [38].

CEA is a cellular adhesion glycoprotein, normally produced exclusively during fetal development [39]. High levels of CEA in the blood are usually associated with subclinical BC metastasis [40].

Despite their utility in BC, these biomarkers exhibit low specificity as they can also increase in benign diseases as well as other malignancies such as gastric, pancreatic, lung, ovarian, colorectal, and liver cancers. Hence, they are not sensitive enough for early detection or routine BC screening [41,42]. Therefore, the utility of these markers is limited to monitoring the response to therapy or disease progression, as indicated by the American Society for Clinical Oncology (ASCO) since 2015 [43].

Another promising diagnostic circulating biomarker is human mammaglobin A (MAM) [44]. MAM is expressed in different tissues, mainly in the breast epithelium but also in the endometrium, sweat glands, and salivary glands [45]. It belongs to the secretoglobins family and has a dimeric structure that binds to lipophilin B [46]. MAM plays a crucial role in BC development, immune system regulation, as well as the transport of steroid hormones and other aromatic molecules [47]. BC cells overexpress this protein, and different assays have been carried out to validate its association with metastasis [48]. Several studies have demonstrated that MAM exhibits good specificity and sensitivity ranging between 77.8 % and 99 % [49,50]. Moreover, MAM seems to be the most accurate serum biomarker for the early detection of BC sentinel lymph node metastasis [51].

While the above-mentioned circulating biomarkers are frequently employed, there is only limited evidence demonstrating their clinical value or capability to improve patients’ outcome. Indeed, most of the current biomarkers exhibit weak specificity and poor sensitivity, particularly for BC early detection and low-volume cancer recurrence [52].

These limitations highlight the unmet need for developing new technologies and strategies for early BC detection and treatment monitoring [53]. The growing body of data suggests how CTCs and ctDNA are more sensitive for early BC detection and recurrent/metastatic disease prediction than standard biomarkers or standard-of-care radiological imaging [54].

## 4 A new frontier for breast cancer biomarkers

### 4.1 CTCs

CTCs are intact, viable, non-hematological nucleated cells with malignant features that are spread from the primary tumor site or from metastatic lesions into the bloodstream as single cells or clusters [55]. However, only a few CTCs survive and infiltrate distant organs, while most of them are lost in blood circulation. Once arrived at distant organs, CTCs become the substrate for metastasis formation [56].

In BC, CTCs are detectable in patients with both early and late stages of the disease [57,58]. CTCs detection is related to clinical outcome and, in particular, CTCs amount before systemic treatment in both metastatic and non-metastatic BC patients seems to be pivotal [59]. CTCs are identified in the bloodstream of approximately 20–50 % of early-stage BC patients, depending on the assay, and about 60 % of those with advanced disease. In early stages, the proposed cut-off is ≥ 1 CTC [60], while in advanced stages, it is ≥ 5, considering the lower detection rate [61]. Furthermore, counting CTCs at different time points may represent a reliable approach during systemic treatment, allowing the characterization of their molecular features, an essential task to optimize therapy [62]. CTCs are indeed a representative fraction of cancer; therefore may be a reliable source of information about tumor biology before and during medical treatments [63,64]. However, due to epithelial-mesenchymal transition (EMT), CTCs differ from primary tumor cells, acquiring properties that allow them to intravasate into the bloodstream, disseminate in clusters, and gain stemness features that enhance their capability to initiate metastasis. The acquisition of a metastatic phenotype by CTCs is largely driven by epithelial-mesenchymal plasticity [65,66].

The detection and isolation of CTCs in BC patients are generally based on the selection of specific epithelial markers, such as EpCAM (mainly), CK8, CK18, and CK19, and the exclusion of leukocytes by CD45, through fluorescence-labeled monoclonal antibodies [57,59,67]. Nowadays, EpCAM-based CTC detection technologies are widely applied for BC [68]; however, they have some limitations: (i) not all BC express EpCAM, therefore it cannot be used in EpCAM-negative or low-expressing tumors [69]; (ii) moreover, different markers, including EpCAM, are down-regulated during EMT, which affects the detection rate of EpCAM-positive CTCs [70]. Currently, alternative commercial platforms to isolate and characterize CTCs are under investigation; however, CellSearch® is still the only FDA-approved platform to predict metastatic BC (mBC) patients outcome [[71], [72], [73]]. The strengths and downsides of CellSearch® are covered in detail below.

CKs have been used as epithelial markers for diagnostic purposes in routine histopathology for over 20 years [74]. CK8, 18, and 19 are generally expressed by luminal cells in normal mammary epithelium. CK8 is a member of the intermediate filaments (IF) gene family and is mostly combined with its partner, CK18, into highly insoluble 10 nm filaments extended from the nucleus to the internal leaflet of the plasma membrane [75]. However, several studies have challenged the notion that CKs are just epithelial markers; indeed, regulatory changes in CKs expression at both the transcriptional and post-transcriptional levels in epithelial cancer cells have been demonstrated [76]. A down-regulation of CK18 was observed in mBC and seems to be predictive of poor clinical outcome. Moreover, the downregulation of luminal CKs (e.g. CK18) in primary tumors is related to the onset of hematogenous metastasis. CK19 is detected in normal and neoplastic epithelial cells as a cytoskeletal component and represents the most sensitive and reliable tumor marker in both early-stage and mBC [77]. Studies have shown that node-negative BC patients with CK19 mRNA-positive CTCs, detected before adjuvant chemotherapy, have shorter PFS and OS [78]. Furthermore, CTCs can express mesenchymal markers if they undergo EMT [79].

The isolation and characterization of CTCs is still a challenging task, at least technically, due to their heterogeneity and very low concentration in the bloodstream [80,81] (see also section 5.1). The heterogeneity of CTCs, which contributes to tumor development, may reflect the plasticity of the disease and its prognosis [82]. CTCs may have different proliferative or apoptotic potentials and various intrinsic molecular profiles. CTCs often do not express ER, PgR, and HER2 and have a low proliferation index, which results in chemotherapy resistance [83].

Therefore, highly specific, and sensitive methods are needed. On the other hand, since cancer cells harbor somatic alterations, including genetic variants, gene amplifications, and chromosomal rearrangements, tumor DNA presents a unique “fingerprint” that can be used to differentiate cancer from non-tumoral cells [84].

### 4.2 ctDNA

ctDNA consists of DNA fragments with tumor-specific alterations [85] and represents a variable fraction of the total circulating cell-free DNA (cfDNA) [86]. Mandel and Métais in 1948 used for the first time the term cfDNA referring to fragmented DNA found in the non-cellular blood component [87]. cfDNA consists of extracellular DNA molecules (double-stranded DNA and mitochondrial DNA) derived from the blood (and other biological fluids) of healthy and unhealthy people [88]. The median concentration of cfDNA in healthy individuals’ plasma is 1–10 ng/mL [89]. Generally, cfDNA levels increase under tissue stressful conditions such as exercise, inflammation or tissue injury (surgery included) [90]. cfDNA molecules originate from hematopoietic cells in healthy individuals and from both normal and tumor cells in cancer patients [91]. However, cfDNA levels in cancer patients have been reported to be significantly higher compared to healthy people [92]. Additionally, the degree of necrosis and apoptosis affects the amount of cfDNA [93]. ctDNA may provide a collective representation of the tumor genome [94], capturing mutations not present in the primary biopsy [95], and helping to overcome issues related to tissue sampling bias [96]. In particular, the analysis of ctDNA provides information about tumor dormancy and highlights its potential use in monitoring BC patients without clinically detectable disease [[97], [98], [99]]. Finally, ctDNA levels may represent a surrogate biomarker to assess the overall residual tumor burden and treatment efficacy after chemotherapy [100,101].

Noteworthy, a fraction of ctDNA is bound to erythrocytes and leukocytes, namely cell-surface-bound circulating DNA (csbDNA). Such interaction could be mediated by the nucleoprotein complexes and might be related to changes in the composition and amount of proteins on blood cell-surface due to cancer [102]. The cellular origin of csbDNA is not yet fully understood. However, like cfDNA, a portion of csbDNA in cancer patients may derive from tumor cells, making it a promising circulating biomarker. Nevertheless, its usefulness is still controversial [103].

Hence, circulating biomarkers, such as CTCs and ctDNA, may become essential in BC diagnosis, as well as prognosis and prediction of therapeutic response, promoting timely identification and appropriate monitoring throughout treatments.

Table 1 summarizes validated and emerging platforms for biomarker detection in BC through tissue and liquid biopsy approaches.

**Table 1:** Comparative overview of current and emerging platforms for breast cancer biomarker assessment across tissue and liquid biopsy samples. The table outlines key parameters including analyte type, sample source, detection scope, advantages, limitations, and clinical applications. Tissue-based assays remain the foundation for diagnostic and molecular characterization, while liquid biopsy methods offer a non-invasive alternative for real-time disease monitoring and therapeutic guidance. Circulating biomarkers, such as ctDNA and CTCs, can be analyzed using various methodologies with varying sensitivity, specificity, and multiplexing capabilities. Each platform is associated with distinct technical features and regulatory status, which determine its clinical utility and integration into standard or investigational workflows.

TECHNOLOGY	ANALYTE	SAMPLE TYPE	DETECTION SCOPE	ADVANTAGES	LIMITATIONS	CLINICAL USE
***FFPE TISSUE***						
**IHC/ISH**	Protein/DNA	FFPE tissue	ER, PR, HER2, Ki-67, PD-L1, PTEN, HER2 amplification	Standard, low cost, widely used	Low multiplexing, difficult standardization	Diagnosis, classification, HER2/HR/Other tissue biomarkers status
**Real-time PCR (qPCR)**	DNA	FFPE tissue	Mutation-specific detection	Fast, cost-effective, widely used	Limited to known variants, lower sensitivity, limited multiplexing	Targeted molecular biomarker status evaluation
**NGS**	DNA/RNA	FFPE tissue	Genomic signature, Somatic BRCA1/2, HRD, TMB, gene fusions, other unknow genomic alteration	Broad profiling, high-throughput, unbiased detection of novel alterations	Require higher yield and good quality genomic material, expensive, higher sensitivity, longer turnaround time	Targeted/untargeted molecular biomarker status evaluation
***LIQUID BIOPSY***						
**Real-time PCR (qPCR)**	ctDNA	Plasma	Mutation-specific detection	Fast, cost-effective, widely used	Limited to known variants, lower sensitivity, limited multiplexing	Targeted molecular biomarker status evaluation
**Digital PCR (dPCR)** **Droplet Digital PCR (ddPCR)**	ctDNA	Plasma	Mutation-specific detection with absolute quantification	High/ultra sensitivity, accurate quantification	Limited to known variants, limited multiplexing	
**BEAMing**	ctDNA	Plasma	Low-frequency mutation-specific detection	Extremely sensitive	Complex workflow, specialized equipment	
**NGS**	ctDNA	Plasma	Mutation-specific detection, SNVs, CNAs, SVs, epigenetic traits, other unknow genomic alteration	Multiplexed, tracks clonal evolution, unbiased detection of novel alterations	Reduced sensitivity in low ctDNA burden, untargeted still expensive and not yet routine	Targeted/untargeted molecular biomarker status evaluation
**CellSearch®**	CTC	Whole blood	Immunomagnetic capture of EpCAM+/CK + cells	FDA-cleared, reproducible, prognostic	Does not detect EMT-CTCs	Prognostic in mBC
**RareCyte®/CyteFinder®/CytePicker®**	CTC	Whole blood	Density and immunofluorescence-based CTCs	Semi-automated, enables multi-marker profiling and single-cell recovery	Requires specialized tubes and equipment	Experimental, longitudinal tracking, EMT-CTCs
**CellSieve™** **ScreenCell®**	CTC	Whole blood	Filtration + cytomorphological and marker ID	Rapid processing; combines morphology and marker detection	Risk of loss of small or deformable CTCs	Experimental, morphologic and phenotypic CTC profiling
**Parsortix™**	CTC	Whole blood	Label-free, size and deformability-based	Captures EMT and EpCAM-low CTCs, viable for downstream analysis	Not diagnostic, enrichment only, downstream validation needed	FDA-cleared for CTC enrichment; functional/omics studies

## 5 Diagnostic purposes

### 5.1 CTCs

Increasing BC screening campaigns have accelerated the identification and validation of diagnostic biomarkers that may improve both prognostic stratification and treatment outcomes [104]. However, the detection of CTCs remains challenging due to their low frequency and the heterogeneous expression of antigens, hampering their use as a diagnostic tool for patients with early-stage BC [105]. In non-metastatic BC, CTCs are particularly rare, usually less than 1 CTC/10 mL of blood [106], and 5 or more CTCs represent a rare event (1–5.9 %) [107]. Nevertheless, using ≥1 CTC/7.5 mL of blood as threshold value, CTCs can be identified in about 20–25 % of patients with BC at the time of diagnosis [108].

### 5.2 ctDNA

While CTCs are mostly present in advanced-stage cancer patients, ctDNA can be easily identified in the majority of early-stage BC patients [109]. Different studies have demonstrated that patients with localized and mBC show ctDNA levels up to approximately five times higher than healthy controls [110,111]**,** demonstrating its diagnostic value.

Molecular-based technologies, including highly sensitive (<0.01 %) digital PCR (dPCR), have been successfully used to detect low concentrations of ctDNA, particularly in patients with early BC. However, establishing a threshold level of ctDNA concentration for BC diagnosis remains challenging due to the wide and overlapped observed ranges [112]. Importantly, high levels of ctDNA are detected regardless of the tumor stage, and cfDNA content may not be specific for BC disease [113].

Currently, aberrantly methylated cfDNA is gaining recognition as a potential diagnostic circulating biomarker [114]. It has been reported that primary breast cancer exhibits hypermethylation of various tumor suppressor genes (see below, paragraph 6.2) [115]. Furthermore, compared to cfDNA, the methylation index of csbDNA seems to be an even more reliable diagnostic tool for BC. Thus, csbDNA could be useful in improving the sensitivity and specificity of LB [102].

## 6 Prognostic purposes

The number of axillary nodal metastases is considered one of the most important prognostic factors in BC [116]. However, relying solely on this feature is not sufficient [117]. While up to 50 % of patients with positive axillary nodes can be cured by local therapy alone (i.e., surgery and radiation) without the need for adjuvant therapy, approximately 30 % of medically untreated BC patients without nodal involvement will experience recurrence in the next decade or more [118]. Hence, new biomarkers are being evaluated to better define the prognosis, as described below [[119], [120], [121], [122]].

### 6.1 CTCs

Significant efforts have been made to evaluate the utility of CTCs in the monitoring of mBC [123]. According to Budd et al. [[124], [125], [126], [127]], the estimation of CTCs has several advantages in monitoring BC beyond traditional imaging methods. Indeed, in 2004, Cristofanilli et al. [57] first demonstrated the prognostic value of CTCs in mBC. In particular, about 60 % of mBC patients showed detectable CTCs levels, and a count of ≥5 CTCs/7.5 mL of blood was associated with significantly worse PFS and OS [128,129].

Though, tumor heterogeneity at metastatic sites encompasses a different level of complexity; indeed, there is an inter-metastatic heterogeneity (between different metastases of the same patient) and an intra-metastasis heterogeneity (between cells within the same metastasis). Early metastases consist of a greater proportion of cells with EMT and ‘stem-like’ features [130], whereas larger metastases consist of a heterogeneous population of cells that closely reflect the heterogeneity of primary tumors [131]. The role of the immune system brings an additional level of complexity since immune destruction of micro-metastases could mean that the ctDNA is primarily a reflection of dead cancer cells [132]; hence, persisting CTCs might be considered a more accurate guide to treatment selection [133,134]: the reduction or absence of CTCs may indicate a positive response to treatment; conversely, the persistence of CTCs may suggest resistance to treatment and an incomplete eradication of the cancer cells, even if no metastasis is clinically detected [[135], [136], [137]].

Recently, the persistence of CK19-positive CTCs has been shown to be related to a lower PFS also in non-metastatic BC settings [138]. Hence, CTCs can provide independent prognostic information, before and after surgery, both in the neoadjuvant and adjuvant settings [139].

Some other studies have suggested a prognostic relevance for CTCs also in patients with triple-negative BC (TNBC), where a high number of CTC was related to lower survival rates [[140], [141], [142]], and in particular, patients with ≥5 CTCs/7.5 mL of blood at baseline exhibit poorer PFS and OS [[143], [144], [145], [146], [147], [148]]. Nevertheless, the distant metastasis rate in TNBC becomes significantly lower after five years of follow-up, probably because dormancy is a less common phenomenon in this BC subtype [149], unlike luminal BC [150,151] where disseminated tumor cells can remain dormant for years and subsequently may result in a metastatic lesion persisting as undetectable minimal residual disease (MRD) [152].

Recent studies have proposed to distinguish between molecular residual disease, detected via ctDNA, and cellular residual disease, identified by CTCs, suggesting that the two may offer complementary insights. While ctDNA provides genomic information with high specificity, CTCs, being intact living cells, allow for multi-omic profiling (DNA, RNA, and protein) and even functional assays, potentially revealing therapy resistance mechanisms and metastatic potential. In particular, in TNBC, persistent CTCs after neoadjuvant treatment were shown to harbor shared genomic alterations with residual tumor, indicating their possible role as treatment-resistant clones. Moreover, combined detection of CTCs and ctDNA has shown improved sensitivity in predicting relapse compared to either marker alone, reaching up to 90 % sensitivity when both were used in tandem.

Overall, LB emerged as a promising strategy for the detection and monitoring of MRD in BC patients. Nevertheless, its clinical implementation remains limited by several unresolved technical and biological challenges [153]. To date, no circulating biomarker has shown the required sensitivity and specificity to be broadly adopted for MRD surveillance.

### 6.2 ctDNA

On the other side, ctDNA is detectable in early and late stages of BC patients [154] and may persist for many years after clinically successful therapy [[155], [156], [157]] without evidence of distant metastases. In mBC, ctDNA seems to be related to serum CA15-3 levels and CTCs counts, which reflect tumor burden changes and treatment response [158], and seems to have higher sensitivity than other circulating biomarkers [159].

Some studies demonstrated the prognostic role of ctDNA in predicting relapse, particularly in high-risk TNBC patients [160,161], and ctDNA levels seem to be associated with patient survival during neoadjuvant chemotherapy [162]. In contrast, other authors did not find a correlation between baseline ctDNA levels and prognosis, pointing to a more significant role for ctDNA in identifying mutations as prospective treatment targets [163].

Current research has shown the prognostic value of ctDNA by the detection of different mutations and DNA methylation [[164], [165], [166], [167], [168]]. Promoter hypermethylation of several genes has been detected in BC tissues, and interestingly, the methylated genes have also been found in ctDNA of 10–30 % of primary BC patients and of 50–80 % of mBC patients. These genes are involved in cell cycle regulation (*p16*^*INK4A*^*, p14*^*ARF*^*, p15, CCDN2, DAPK*), DNA repair (*BRCA1, MGMT, hMLH1*), detoxification (*GSTP1*), signal transduction (*RARβ2, APC, Erβ*), adhesion and metastasis (*CDH1, CDH13*) [169]. DNA methylation provides significant advantages over mutation detection for ctDNA analysis [170,171]. Strong results have demonstrated how cancer development involves both genome-wide hypomethylation and gene-specific hypermethylation [[172], [173], [174], [175], [176]]. The detection of tumor-specific DNA methylation in serum has currently proved to be useful in prognostication and in monitoring therapeutic responses in BC patients [177]. Indeed, BC patients with methylated-ctDNA showed a poorer prognosis [[178], [179], [180], [181], [182], [183], [184]] and a biologically more aggressive disease. Furthermore, the presence of methylated ctDNA could be related to the presence of CTCs [[185], [186], [187], [188]]. Lastly, methylated ctDNA in BC patients treated with neoadjuvant chemotherapy is related to pathological response [[189], [190], [191], [192]].

## 7 Predictive purposes and treatment monitoring

CTCs and ctDNA are arguably the best real-time LB markers for disease monitoring and therapy selection. Moreover, both CTCs and ctDNA, which provide different but complementary information, have been associated with patients’ survival and treatment response [[193], [194], [195], [196]].

### 7.1 CTCs

The predictive value of CTCs has yet to be proven in early BC; in patients with advanced BC, instead, Hayes et al. demonstrated that HER2-positive cells can be quantified through CTCs [197]. Intriguingly, the authors discovered a minority of patients who had a phenotypic conversion from HER2-negative to HER2-positive CTCs during treatment [198]. Muller et al. [199] confirmed these results in a large multi-institutional analysis with nearly 2000 patients enrolled, demonstrating the strong prognostic impact of CTCs detection and highlighting how patients with HER2-negative BC but HER2-positive CTCs may benefit from HER2-targeted therapy. Moreover, HER2-positive CTCs appear to be more prevalent in patients with mBC [200]. These findings imply that HER2 expression exerts a biological impact on CTCs, indicating that alterations in the HER2 status of CTCs may play a role in BC progression and the development of secondary drug resistance [201]. Some authors pinpointed that patients who acquired HER2 overexpression in CTCs had primary HR-positive BC pretreated with concomitant chemo- and endocrine therapy [[202], [203], [204], [205]]. These data suggest that HER2 conversion can occur during endocrine treatment and could be related to a cancer adaptation mechanism to survive, or to an ER-induced modulation of HER2 expression triggered by endocrine therapy [206,207]. Finally, molecular discrepancies between primary tumor and CTCs, not only related to HR and HER2, have been reported, such as in MHC III and EGFR [[208], [209], [210], [211]].

Recent studies have shown that CTCs can help guide the selection of chemotherapy or endocrine therapy in the first-line treatment of HR-positive/HER2-negative breast cancer. This demonstrates that identifying CTCs may expand the number of patients eligible for endocrine therapy and could provide insight into why endocrine therapy fails in a subset of primary HR-positive BC patients [212,213].

Notably, the STIC-CTC trial demonstrated that CTC enumeration can outperform clinical risk assessment in determining treatment strategy, with improved survival outcomes observed in patients classified as clinically low-risk but CTC-high, who benefited from chemotherapy rather than endocrine therapy alone [214]. Similarly, exploratory findings from the PACE trial supported the use of CTC count to identify patients with endocrine-resistant disease who may benefit from treatment escalation, including immunotherapy [215]. Furthermore, the DETECT III trial provided the first evidence that CTC phenotyping may guide targeted therapy, showing that patients with HER2-negative mBC but HER2-positive CTCs had significantly improved survival when treated with lapatinib in addition to standard therapy [216].

### 7.2 ctDNA

ctDNA fragments are easily detectable and have a short half-life, which makes ctDNA a useful method to investigate treatment-related tumor changes and monitor drug response in a real-time clinical setting. Indeed, ctDNA monitoring is an accurate method for the detection of occult metastasis, and the sequencing of ctDNA sampled during treatment has been shown to provide pivotal monitoring information [217]. In addition, ctDNA levels, even when measured in the setting of primary (non-metastatic) BC, are associated with poor outcome [218]. By contrast, CTCs are not recommended for routine monitoring after primary surgery [219,220]. To improve sensitivity in detecting MRD in primary BC, patient-specific sequencing assays have been most commonly employed [221]. Indeed, several proof-of-concept studies have demonstrated the utility of ctDNA as an early marker of therapeutic resistance and MRD [222]. However, this approach presents limitations, especially in BC cases characterized by high intratumoral genetic heterogeneity. This is because the genomic alterations used to monitor the disease are restricted to a subpopulation of cells and may not be representative of the entire tumor. Conversely, recent evidence supports the use of ctDNA as a reliable biomarker for monitoring tumor burden dynamics in mBC patients undergoing systemic therapy [223,224].

Different circulating blood biomarkers (CTCs and CA 15-3) and tumor imaging were shown to be less sensitive and specific than ctDNA. Additionally, a strong correlation was observed between the number of amplifiable ctDNA copies and patient prognosis, with fluctuations in ctDNA levels reflecting treatment response [[225], [226], [227]].

Furthermore, HER2 gene detection in ctDNA can be leveraged to assess its status during treatment, serving as a complementary method to tumor tissue biopsy. Studies have shown that cfDNA levels and HER2 gene amplification in cfDNA increase during neoadjuvant chemotherapy, although no direct correlation with treatment efficacy has been observed [228]. Notably, Page et al. [229] demonstrated the presence of HER2-amplified ctDNA in both disease-free and mBC patients, with high concordance to the primary tumor. These findings suggest a potential predictive and prognostic role for ctDNA in the HER2-positive BC setting.

With the introduction of poly ADP ribose polymerase inhibitors (PARPi), the use of ctDNA in TNBC has evolved [230]. Germline BRCA mutations are around 5 % in all BCs and 16–40 % in TNBC patients [231,232]. These mutations compromise the homologous recombination repair (HRR), an accurate mechanism that helps repair damaged DNA [233]. By suppressing DNA single-strand break repair, PARPi promote the accumulation of DNA replication errors in BC and cell cycle arrest. PARPi have been shown to improve patient outcomes in BC with germline BRCA mutations, irrespective of tumor HR/HER2 status [234,235]. Resistance to PARPi is due to secondary intragenic deletions or reverse mutations, which restore the open reading frame of a germline BRCA mutation, leading to a functional HRR. Once identified in ctDNA, these mutations could serve as predictive markers for resistance to PARPi, highlighting the potential role of ctDNA in selecting patients [[236], [237], [238], [239]].

Finally, both preclinical and clinical studies have highlighted that one of the most common mechanisms of endocrine treatment resistance in HR+/HER2-BC is the presence of activating mutations in the estrogen receptor alpha gene (*ESR1*). These mutations, which are associated with therapeutic resistance, can be detected in ctDNA [[240], [241], [242]]. BC patients with ESR1-mutant ctDNA have poorer outcomes, and *ESR1* mutations are common in metastatic HR+ BC patients treated with aromatase inhibitors (AI). Hence, ctDNA with *ESR1* mutations can be considered a marker of aggressive disease and can guide further therapies, as noted below [227]. The EMERALD trial (NCT03778931) first demonstrated that elacestrant, an oral selective estrogen receptor degrader (SERD), significantly improved PFS compared to standard endocrine therapy in patients with ESR1-mutant HR+/HER2− mBC previously treated with CDK4/6 inhibitors [243]**.** These results led to FDA approval of elacestrant alongside the Guardant360 CDx ctDNA assay as a companion diagnostic. In contrast, the PADA-1 trial (NCT03079011) adopted a proactive approach, enrolling patients receiving first-line AI plus CDK4/6 inhibitor therapy. Through serial monitoring of ctDNA, *ESR1* mutations were detected before clinical progression, allowing for early therapeutic intervention [244]. Patients were randomized to continue AI or switch to fulvestrant, both in combination with palbociclib. The early switch significantly improved PFS. More recently, the SERENA-6 trial (NCT04964934) further validated this proactive strategy by showing that early switching to camizestrant, a next-generation oral SERD, upon detection of emergent ESR1 mutations in ctDNA during AI plus CDK4/6i therapy, significantly prolonged PFS compared to continuing AI [245]. Notably, SERENA-6 is the first registration-phase trial to prospectively incorporate ctDNA dynamics to guide treatment modification in the absence of radiographic progression. Together, EMERALD, PADA-1, and SERENA-6 exemplify a continuum of ctDNA-based strategies: EMERALD supports ctDNA as a tool for selecting targeted therapies after progression, while PADA-1 and SERENA-6 reinforce its role in guiding preemptive treatment adaptation, underscoring the expanding clinical relevance of liquid biopsy in endocrine-resistant HR+/HER2− BC.

Finally, although most mutation analyses rely on LB, emerging evidence suggests that FFPE metastatic tissue samples may serve as a viable alternative or complementary source for mutation detection, particularly when ctDNA testing is unavailable or unfeasible [246] (see also Fig. 2).

**Fig. 3:**
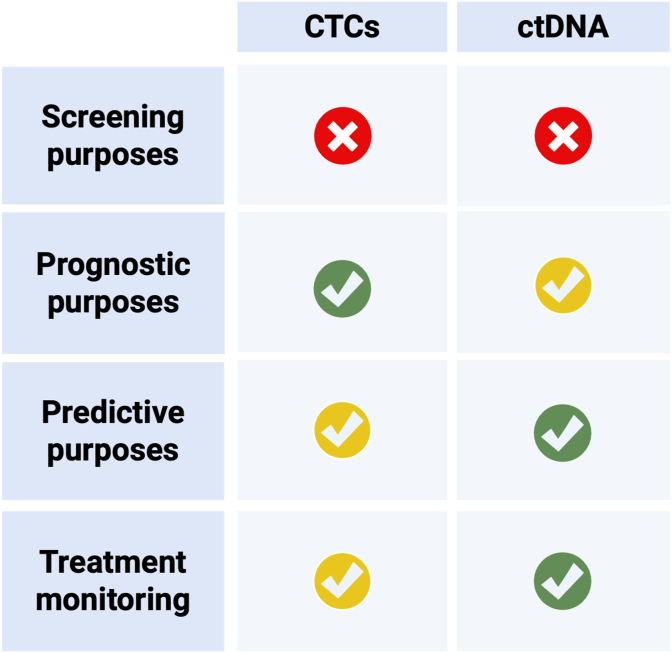
Schematic representation of the clinical utility of circulating tumor DNA (ctDNA) and circulating tumor cells (CTCs) in breast cancer, categorized by level of evidence: green indicates validated and approved applications, yellow indicates strong but still experimental evidence, and red indicates areas with insufficient clinical evidence. *Created with* BioRender.com.

Fig. 3summarizes the applications and limitations of CTCs and ctDNA in different settings.

## 8 CTCs and ctDNA in real-life scenarios: from FDA approvals to clinical trials

### 8.1 FDA-approved assays

The great interest and the promising results of LB in several retrospective and prospective trials conducted over the last years led to the approval of several blood tests based on CTCs or ctDNA by the FDA, mainly regarding the non-invasive management of advanced cancers.

#### 8.1.1 CTCs

In the early 2000s, the FDA approved the CellSearch System® platform (Veridex, Raritan, NJ, USA) for prognostic use, the first LB assay designed for CTCs detection in breast, colorectal, and prostate cancer patients. CellSearch System® detects and enumerates CTCs of epithelial origin using antibodies against CD45, EpCAM, and cytokeratins 8, 18, and/or 19, from a 7.5-mL blood sample [247,248]. In several clinical trials, CTCs measured by the CellSearch® proved to be a strong, independent predictor of OS and PFS in all the above-mentioned cancers.

In BC patients, the value of ≥5 CTCs in 7.5 ml blood samples was associated with worse OS and PFS [57]. In another study by Mu et al., the positive baseline count of CTCs in the blood sample of BC patients detected with the CellSearch® correlated with a shorter PFS. In addition, CTCs clusters seem to provide additional prognostic value for their increased presence in inflammatory BC [249,250]. Other subsequent trials had similar results, demonstrating that the CTCs number assessed through the CellSearch® platform was positively correlated with a worse prognosis [251,252].

The predictive role of CTCs in mBC patients was assessed by Jakabova et al. They used the CellSearch System® platform to monitor the response to palbociclib in 46 patients with advanced BC, showing that patients with detectable CTCs after the first cycle of palbociclib had a shorter PFS [253].

Nevertheless, there are several concerns regarding the application of the CellSearch® platform. The first issue is related to the use of fixatives to keep whole blood stable for up to 96 h for short-term storage and transportation. Indeed, fixation compromises the viability of the cell and the RNA integrity. Thus, it is not possible to use the sample for downstream applications, such as xenograft modeling, *ex vivo* culture creation, and gene expression analysis [[254], [255], [256]]. Other important concerns regard the limited amount of CTCs that can be detected in the bloodstream of cancer patients, which impacts the accuracy of their assessment for several clinical purposes, such as the evaluation of MRD after surgery or prediction of early relapse [106]. This is clearly demonstrated in a study involving 1087 early-stage high-risk BC patients, which shows that although CTCs detected two years after the completion of chemotherapy still retain prognostic significance, the sensitivity of a positive CTC status for predicting disease relapse remains low (36 %) [257].

Although the use of the CellSearch® technology is considered the gold standard for CTCs enumeration, other technologies have been developed and demonstrated similar prognostic value despite the EpCAM status. Promising results in detecting CTCs were obtained by the RareCyte platform, and by two other technologies: CellSieve™ and ScreenCell® [[258], [259], [260]]. Briefly, the RareCyte platform, using the AccuCyte® kit, specialized blood tubes, and collecting devices, quickly identifies CTCs depending on density. First, the red blood cell fraction is divided into nucleated and non-nucleated blood cells. The nucleated fraction is then distributed among eight conventional glass slides that can be stained with a maximum of six fluorescent markers. The CyteFinder® instrument, a digital scanning microscope, scans these slides and performs a semi-automatic analysis. Finally, a fluid‐coupled picking system placed above the slide stage is used to mechanically retrieve CTCs.

CellSieve™ and ScreenCell® are very similar and employ an enrichment strategy based on short-time filtration through a membrane, but use different criteria for tumor cells identification. The first one is based on marker expression like CellSearch®, whereas the latter is based on the cytomorphological evaluation.

Another widely used platform is Parsortix™ (Angle plc), a label-free microfluidic device that isolates CTCs based on physical properties such as size and deformability, rather than surface marker expression. Blood flows through a cassette containing a stepped narrowing channel, which captures larger and less deformable CTCs while allowing smaller blood cells to pass through. This method enables enrichment of EpCAM-low or EpCAM-negative CTCs, such as those undergoing EMT, which are often missed by epithelial marker-dependent platforms. Parsortix™-isolated CTCs are viable and compatible with downstream molecular analyses, including RNA-seq and single-cell genomic profiling, making it particularly suited for functional and translational research applications. Parsortix™ is FDA-cleared for CTC enrichment, but not for diagnostic use; isolated cells must be analyzed using separately validated downstream assays.

#### 8.1.2 ctDNA

Promising results have demonstrated the strong association between tumor burden and ctDNA and its potential role to guide and personalize the choice of molecularly targeted therapies [261]. Cell-free DNA analysis using NGS is a recent tool introduced in clinical practice, and it is an informative, highly specific, and sensitive biomarker in metastatic disease. In fact, the FDA has approved a single-gene test and two multigene assays as companion diagnostics to identify patients who may benefit from specific treatments by detecting genomic alterations in plasma cfDNA of several solid tumors, including BC.

The *Therascreen PIK3CA RGQ PCR* kit is a PCR-based test for the detection of 11 mutations in the *PIK3CA* gene using genomic DNA (gDNA) from BC tissue or ctDNA. If a molecular alteration in this gene is detected, on the basis of SOLAR-1 trial results [262], the FDA approves the administration of alpelisib, a PI3Kα inhibitor, in association with fulvestrant in advanced and metastatic HR+/HER2- BC.

In August 2020, the Guardant360 CDx assay was the first approved by the FDA as a qualitative NGS-based *in vitro* diagnostic device for the simultaneous detection of point mutations (SNVs) or deletions variants, amplifications, and fusions in tumor-associated genes using plasma cfDNA samples. This LB CGP test was originally approved for the identification of *EGFR* mutations in patients with non-small cell lung cancer (NSCLC), in particular deletions in exon 19, L858R in exon 21, or T790M in exon 20, to predict the benefit from osimertinib. The Guardant360 CDx assay was later approved as a complementary diagnostic to assess the mutation profiles of patients with any form of solid cancer. In particular, resistance to treatment with alpelisib was observed in mBC patients with mutations in *PTEN* and *ESR1* detected by Guardant360 assay [[263], [264], [265]]. On January 27, 2023, FDA approved the Guardant360 CDx assay as a companion diagnostic device to identify patients with ER-positive/HER2-negative and *ESR1*-mutated advanced or mBC for treatment with Elacestrant, the first oral selective ER degrader demonstrating a significant PFS improvement versus Standard Of Care (SOC) [266,267].

FDA also approved FoundationOne® Liquid CDx, which detects substitutions, insertions, and deletions (indels), rearrangements and copy number alterations in tumor-associated genes using plasma cfDNA samples. Albeit this test was approved as a complementary diagnostic for tumor mutation profiling in patients with NSCLC and prostate cancer, its use is currently extended to breast and ovarian cancer. In mBC, this assay is valuable for identifying patients with specific *PIK3CA* mutations, which can predict their response to alpelisib treatment. The absence of cfDNA genomic findings does not necessarily indicate their lack in the corresponding tumor tissue; for this reason, in case of a negative cfDNA test, patients should be reflexed to routine tumor biopsy samples using an FDA-approved assay [268].

In 2019 The New York State Department of Health approved the *Analysis of Circulating Cell-free DNA to Evaluate Somatic Status* (MSK-ACCESS), a LB assay developed by the researchers of the Memorial Sloan Kettering Cancer Center for the detection of low-frequency somatic alterations in 129 genes. This cfDNA test uses hybridization capture and deep sequencing to identify all classes of somatic genetic alterations, including SNVs, indels, copy number alterations, and structural variants [269,270].

### 8.2 CTCs and ctDNA in ongoing trials: a glimpse of future possible applications

So far LB has been implemented in clinical practice only for a few specific clinical purposes regarding the management of metastatic disease. The reasons behind such limited impact rely on the suboptimal accuracy of CTCs and ctDNA in the assessment of cancers with a low burden of disease, the lack of standardization in the assessment of such biomarkers, and the challenges of integrating such assays into clinical workflows due to high costs and infrastructure limitations [271,272].

Nevertheless, the assessment of CTCs or ctDNA has been stably implemented in the design of clinical trials together with innovative approaches to identify novel biomarkers [[273], [274], [275], [276], [277], [278], [279], [280], [281], [282]], dissecting tumor microenvironment [[283], [284], [285], [286]] and cancer secretome in the bloodstream, such as the study of cfDNA methylation with novel enrichment-based methods [22,287,288].

In particular, the assessment of either CTCs or ctDNA: (i) is nearly a ubiquitous exploratory endpoint in oncology trials for their value as biological correlates of clinically meaningful endpoints, such as OS or PFS; (ii) has been proposed as the main criteria for patients’ randomization or allocation in several clinical trials of escalation or de-escalation strategies; (iii) has been proposed as a primary endpoint for assessing the response to therapeutic interventions.

While the description of ongoing observational trials aimed at evaluating CTCs or ctDNA as a primary or exploratory aim is outside the purpose of this review, we report in Table 2, Table 3 the ongoing trials in which patients’ randomization or allocation to therapy is secondary to CTCs or ctDNA assessment or in which CTCs or ctDNA represent the main outcome measure for assessing the response to treatment.

**Table 2:** Ongoing trials in which patients’ randomization or therapy allocation is secondary to CTCs identification and characterization or CTCs represent the outcome measure for the assessment of the response to therapy.

NCT Number	Title	Type	Status	Intervention	Population	Setting	Biomarker Usage	Estimated Enrollment patients
NCT04993014	Circulating Tumor Cells and Treatment De-escalation After Neoadjuvant Therapy for HER2 Positive Breast Cancer	Interventional (Phase II)	Recruiting	Cohort A (HER2+ CTCs)	Patients with HER2+ BC	Adjuvant chemotherapy	Randomization criteria	80
				Arm A: Trastuzumab				
				Arm B: Trastuzumab + Pertuzumab				
				Cohort B (HER2- CTCs)				
				Arm A: Trastuzumab				
				Arm B: Trastuzumab + Pertuzumab				
NCT04902937	Association of Adjuvant Radiotherapy of Non-metastatic Breast Carcinoma With Immunomodulation and Circulating Tumor Cell Phenotype in Relation to Patient Age	Observational	Recruiting	/	Patients with Localized BC undergoing breast-conserving surgery followed by adjuvant radiation therapy	Adjuvant radiotherapy	Outcome measure	200
NCT04065321	Circulating Tumor Cell Detection in Patients With Luminal A Breast Cancer	Observational	Recruiting	Control group: PET-CT exam	Patients with Localized Luminal A BC	Early detection of relapse	Allocation criteria	500
				Trial group: CTC assessment followed by PET-CT in case of positivity				
NCT04059003	CTC Changes and Efficacy of Neoadjuvant Chemotherapy for Triple-negative Breast Cancer	Observational	Recruiting	/	Patients with triple negative BC undergoing neoadjuvant chemotherapy	Neoadjuvant chemotherapy	Outcome measure	200
NCT03213041	Pembrolizumab and Carboplatin in Treating Patients With Circulating Tumor Cells Positive Metastatic Breast Cancer	Interventional (Phase II)	Recruiting	Single arm: Pembrolizumab and Carboplatin in patients with CTCs	Patients with HER2- metastatic BC	Metastatic disease	Inclusion criteria	100
NCT02035813	DETECT IV - A Study in Patients With HER2-negative Metastatic Breast Cancer and Persisting HER2-negative Circulating Tumor Cells (CTCs).	Interventional (Phase II)	Active, Not Recruiting	Arm A: Ribociclib in combination with standard endocrine therapy (HR + HER- BC)	Patients with HER2- metastatic BC	Metastatic disease	Randomization criteria	116
				Arm B: Eriubulin (HR + HER2- BC and Triple Negative BC)				
NCT06067503	Biomarkers to Detect Endocrine Therapy Resistance	Interventional (Phase II)	Recruting	Single arm: 18F-fluorofuranylnorprogesterone (ER/PR + BC)	Patients with metastatic ER/PR + BC	Metastatic disease	Outcome measure	8

**Table 3:** Ongoing trials in which patients’ randomization or therapy allocation is secondary to ctDNA isolation and characterization or ctDNA represents the outcome measure for the assessment of the response to therapy.

NCT Number	Title	Type	Status	Intervention	Population	Setting	Biomarker Usage	Estimated Enrollment pts
NCT02965755	Personalized Molecular Profiling in Cancer Treatment at Johns Hopkins	Interventional	Recruiting	Assessment of ctDNA and CTC for treatment personalization	Patients with metastatic BC	Metastatic disease	Outcome measure	200
NCT05625087	Detection of Tumor DNA in the Blood of Patients Receiving Standard Therapy for Hormone Receptor-positive (HR+) Non- HER2 Expressing (HER2-) Metastatic Breast Cancer as a Tool to Select Those Who May Benefit From the Next Course of Fulvestrant in Combination with Alpelisib or Ribociclib	Interventional (Phase II)	Not yet Recruiting	Arm A: Alpelisib and fulvestrant Arm B: Ribociclib and fulvestrant	Patients with HR+, HER2- metastatic BC with PIK3CA mutation in ctDNA	Metastatic disease	Randomization criteria	162
NCT04985266	A Trial of Early Detection of Molecular Relapse With Circulating Tumour DNA Tracking and Treatment With Palbociclib Plus Fulvestrant Versus Standard Endocrine Therapy in Patients With ER Positive HER2 Negative Breast Cancer	Interventional (Phase II)	Recruiting	Arm A: Endocrine therapy Arm B: Palbociclib and fulvestrant	Patients with HR + HER2- BC	Adjuvant chemotherapy	Randomization criteria	1100
NCT04920708	Fulvestrant, Ipatasertib and CDK4/6 Inhibition in Metastatic ER+/HER2- Breast Cancer Patients Without ctDNA Suppression	Interventional	Not yet Recruiting	Arm A (high ctDNA): Palbociclib + Fulvestrant + Ipatasertib Arm B high (ctDNA+): Palbociclib + Fulvestrant Arm C (ctDNA-): Standard of Care Arm D (low ctDNA): Standard of Care	Patients with HR + HER2- metastatic BC	Metastatic disease	Randomization criteria	324
NCT04915755	Efficacy and Safety Comparison of Niraparib to Placebo in Participants With Human Epidermal Growth Factor 2 Negative (HER2-) Breast Cancer Susceptibility Gene Mutation (BRCAmut) or Triple-Negative Breast Cancer (TNBC) With Molecular Disease	Interventional (Phase III)	Recruiting	Arm A: Niraparib Arm B: Placebo	Patients with BRCA mutated BC or triple negative BC	Adjuvant chemotherapy	Randomization criteria	800
NCT04849364	Circulating Tumor DNA Enriched, Genomically Directed Post-neoadjuvant Trial for Patients With Residual Triple Negative Breast Cancer	Interventional (Phase II)	Recruiting	Arm 1a (ctDNA with genomic target): talazoparib + capecitabine Arm 1b (ctDNA with genomic target): atezolizumab + capecitabine Arm 1c (ctDNA with genomic target): inavolisib + capecitabine followed by atezolizumab Arm 1d (ctDNA with genomic target): talazoparib + atezolizumab + capecitabine Arm 2 (ctDNA with no genomic target): capecitabine and/or pembrolizumab Arm 3 (ctDNA-): capecitabine and/or pembrolizumab	Patients with Triple negative BC with residual disease at surgery	Adjuvant chemotherapy	Randomization/Allocation criteria	197 patients
NCT04803539	A Prospective, Phase II Trial Using ctDNA to Initiate Post- operation Boost Therapy After Adjuvant Chemotherapy in TNBC	Interventional (Phase II)	Not yet Recruiting	Arm A: Capecitabine Arm B: Capecitabine + Apatinib + Camrelizumab	Patients with triple negative BC	Adjuvant chemotherapy	Randomization criteria	260
NCT04720729	Chemotherapy Monitoring by ctDNA in HER2- Metastatic Breast Cancer	Interventional (Phase II)	Recruiting	Treatment personalization upon ctDNA assessment	Patients with HER2- metastatic BC	Metastatic disease	Allocation criteria	214
NCT04567420	DNA-Guided Second Line Adjuvant Therapy For High Residual Risk, Stage II-III, Hormone Receptor Positive, HER2 Negative Breast Cancer	Interventional (Phase II)	Recruiting	Arm A: Palbociclib/Fulvestrant Combination Arm B: Standard of care	Patients with HR + HER2- BC	Adjuvant chemotherapy	Randomization criteria	100
NCT04501523	A Prospective, Phase II Trial Using ctDNA to Initiate Post- operation Boost Therapy After NAC in TNBC	Interventional (Phase II)	Recruiting	Arm A (ctDNA+, non-pCR): Tislelizumab and Capecitabine Arm B: (ctDNA+, non-pCR): Capecitabine Arm C: (ctDNA+, pCR): Capecitabine Arm D: (ctDNA-): Standard care	Patients with triple negative BC	Adjuvant chemotherapy	Randomization/Allocation criteria	460
NCT04434040	Atezolizumab + Sacituzumab Govitecan to Prevent Recurrence in TNBC (ASPRIA)	Interventional (Phase II)	Recruiting	Atezolizumab and Sacituzumab Govitecan	Patients with triple negative BC	Adjuvant chemotherapy	Outcome measure	40
NCT04256941	Aromatase Inhibitor Therapy With or Without Fulvestrant for the Treatment of HR Positive Metastatic Breast Cancer With an ERS1 Activating Mutation, the INTERACT Study	Interventional	Recruiting	Arm A: ribociclib, palbociclib, and/or abemaciclib + fulvestrant Arm B: ribociclib, palbociclib, and/or abemaciclib, letrozole + letrozolo or anastrozolo	Patients with HR + metastatic BC	Metastatic disease	Inclusion criteria/biomarker of response	124
NCT04059003	CTC Changes and Efficacy of Neoadjuvant Chemotherapy for Triple-negative Breast Cancer	Observational	Recruiting	Arm A: Taxanes or/and anthracycline-based Arm B: Taxanes or/and anthracycline-based	Patients with triple negative BC	Neoadjuvant chemotherapy	Outcome mesure	200
NCT03881384	Circulating Tumor DNA as Marker of Therapeutic Efficacy in Breast Cancer Patients	Observational	Recruiting	/	Patients with localized BC	Neoadjuvant chemotherapy	Outcome measure	200
NCT03213041	Pembrolizumab and Carboplatin in Treating Patients With Circulating Tumor Cells Positive Metastatic Breast Cancer	Interventional	Recruiting	Arm A: pembrolizumab + carboplatin	Patients with triple negative metastatic BC	Metastatic disease	Inclusion criteria	100
NCT03145961	A Trial Using ctDNA Blood Tests to Detect Cancer Cells After Standard Treatment to Trigger Additional Treatment in Early Stage Triple Negative Breast Cancer Patients	Interventional	Active, not Recruiting	Arm A: observational Arm B: pembrolizumab every 3 weeks	Patients with triple negative BC	Adjuvant chemotherapy	Randomization criteria	208
NCT03079011	Palbociclib and Circulating Tumor DNA for ESR1 Mutation Detection	Interventional	Active, not Recruiting	STEP 1: Palbociclib + Aromatase Inhibitors STEP 2 Arm A: no change of therapy STEP 2 Arm B: palbociclib + fulvestrant STEP 3 (cross over): fulvestrant + palbociclib	Patients with ER + HER2- metastatic BC	Metastatic disease	Inclusion/Randomization criteria	1017
NCT02913430	Treatment of Metastatic Breast Cancer With Fulvestrant Plus Palbociclib or Tamoxifen Plus Palbociclib	Interventional	Active, not Recruiting	Arm A: Fulvestrant + palbociclib Arm B: Tamoxifene + palbociclib	Patients with ER + metastatic BC	Metastatic disease	Outcome measure	150
NCT06087120	Investigate the Prognostic and Predictive Value of ctDNA During Neoadjuvant Chemotherapy for Breast Cancer.	Observational	Recruiting	Neoadjuvant chemotherapy (NAC)/treatment regimens for stage II-III HER+/Triple Negative BC	Patients with stage II-III HER+/Triple Negative BC	Neoadjuvant treatment	Outcome measure	75
NCT06517212	Tirzepatide Weight Loss for MRD + Early Breast Cancer (TRIM-EBC)	Interventional (Phase II)	Recruiting	Single arm: Tirzepatide	Patients with a diagnosis of hormone receptor-positive (ER+ > 10 %), and HER2-negative BC within the past 15 years with obesity or overweigh	Adjuvant treatment	Outcome measure	48
NCT05058183	Safe De-escalation of Chemotherapy for Stage 1 Breast Cancer	Interventional	Recruiting	ctDNA test after surgery	Stage 1 HER2 positive and triple negative BC	/	Outcome measure	400
NCT06401421	EXActDNA-003/NSABP B-64: Study of Molecular Residual Disease Detection in Breast Cancer (MRD)	Observational	Recruiting	Diagnostic Test: ctDNA MRD test	High Risk Early BC	/	Outcome measure	1800
NCT06613516	Effect of Capivasertib on ctDNA in ER Positive Breast Cancer (CaptAin)	Interventional (Phase II)	Recruiting	ctDNA dynamics during treatment with Capivasertib	Early stage (I-III) ER positive HER2 negative BC	/	Outcome measure	19
NCT06666439	Longitudinal Tumor Burden Quantification Using Circulating Tumor DNA in Metastatic Lobular Breast Cancer (LBC-Monitor)	Observational	Recruiting	ctDNA change in patients receiving first line endocrine therapy	Metastatic lobular BC with ER+ and HER2-negative	Metastatic disease	Outcome measure	20
NCT05770531	Circulating Tumor DNA to Guide Changes in Standard of Care Chemotherapy	Interventional (Phase II)	Recruiting	Arm A: Standard of care Arm B: standard of care or sacituzumab govitecan IV based on ctDNA results on study	Metastatic estrogen receptor (ER), PR, HER2 negative invasive BC	Metastatic disease	Randomization criteria	120
NCT05708235	A PoC Study to Evaluate Treatments' Efficacy by Monitoring MRD Using ctDNA in HR-positive/HER2-negative EBC Population (MiRaDoR)	Interventional (Phase II)	Recruiting	Arm A: control arm Arm B: Experimental Arm with giredestrant Arm C: Experimental Arm with giredestrant + abemaciclib Arm D: Experimental Arm with giredestrant + inavolisib	HR-positive/HER2-negative early-stage BC at higher risk of relapse	Adjuvant	Inclusion/randomization criteria	1260
NCT06535893	Sustainable and Efficient Platform Trial of New Therapeutic Development for Early Breast Cancer	Interventional (Phase II)	Recruiting	Arm A: Carboplatin + paclitaxel + pembrolizumab followed by doxorubicin + cyclophosphamide + pembrolizumab Arm B: Carboplatin + paclitaxel + pembrolizumab followed by niraparib + pembrolizumab Niraparib Pembrolizumab AC	Early-stage BC (stage II-III)	Neo-adjuvant	Outcome measure	100
NCT06643585	A Randomized Secondary Adjuvant Treatment Intervention Study Comparing Trastuzumab-Deruxtecan to SOC Therapy in EBC Patients with Molecular Relapse (SURVIVE HERoes)	Interventional (Phase III)	Recruiting	Arm A: Trastuzumab-Deruxtecan + endocrine therapy for 16 cycles or until relapse, if earlier Arm B: Continuous treatment of physician's choice	Intermediate to high-risk (as defined in the SURVIVE trial) HER2-positive or HER2-low early BC	Adjuvant	Randomization criteria	180
NCT05826964	Levels of Circulating Tumor DNA as a Predictive Marker for Early Switch in Treatment for Patients With Metastatic (Stage IV) Breast Cancer	Interventional (Phase II)	Recruiting	Step 1: All patients will be receiving standard of care frontline treatment regimens. Step 2: A subset of patients in Step 1 will be randomized to continue same treatment (Arm 1) or switch to new treatment (Arm 2). Step 3: A subset of patients in Arm 1 will be switched to new treatment at time of clinical disease progression	ER+, HER2- metastatic BC	Metastatic	Randomization criteria	500
NCT06067061	neoBREASTIM": Atezolizumab Plus RP1 Oncolytic Immunotherapy in the NeoAdjuvant Setting of Triple-Negative Breast Cancer (neoBREASTIM)	Interventional (Phase I/II)	Recruiting	Single arm: Atezolizumab + RP1	Advanced TNBC	Metastatic	Randomization criteria	51
NCT06230185	CtDNA Based MRD Testing for NAC Monitoring in TNBC (B-STRONGER-I)	Observational	Recruiting	Correlation of molecular residual disease detection by NeXT Personal CTA to pathological Complete Response after neoadjuvant chemotherapy	Stage I-III triple negative BC	Neoadjuvant	Outcome measure	422
NCT05512364	Elacestrant for Treating ER+/HER2- Breast Cancer Patients With ctDNA Relapse (TREAT ctDNA)	Interventional (Phase III)	Recruiting	Arm A: Elacestrant Arm B: standard endocrine treatment - the same they were receiving at the time of ctDNA detection	ER+/HER2- BC and ctDNA relapse.	ER + patients with BC with no evidence of metastasis	Inclusion criteria	220
NCT05959291	Discontinuation of Maintenance HER-2 Directed Therapy in Long-Term Survivors of Metastatic HER-2 Positive Breast Cancer (Free-HER)	Interventional	Recruiting	Discontinuation of Anti-HER-2 Maintenance Treatment	HER-2 positive metastatic (Stage IV) BC	Metastatic	Inclusion criteria	20
NCT05935384	SIBYL: obServation of Therapy Response With lIquid BiopsY evaLuation	Observational	Recruiting	Guardant 360	Unresectable Stage III/IV HR + HER2- BC	Diagnostic	Outcome measure	440
NCT06227728	Analysis of PD-L1, TMB, MSI and ctDNA Dynamics to Predict and Monitor Response to Immunotherapy in Metastatic Cancer.	Observational	Recruiting	IV stage BC indicated for ICI	Metastatic	/	Outcome measure	50
NCT05982678	Basket Study for Oligo-metastatic Breast Cancer (ANISE)	Interventional (Phase II)	Recruiting	Trastuzumab-deruxtecan	Oligo-metastatic HER2 positive BC	Metastatic	Outcome measure	72
NCT03079011	Safety and Efficacy of Palbociclib in Combination with HT Driven by CtDNA ESR1 Mutation Monitoring in ER+, HER2- Metastatic BC Patients (PADA-1)	Interventional (Phase III)	Complete	Arm A: Palbociclib + Aromatase inhibitor Arm B: Palbociclib + fulvestrant	ER+/HER2- BC	metastatic	Randomization criteria	1017
NCT03778931	Elacestrant Monotherapy vs. Standard of Care for the Treatment of Patients With ER+/HER2- Advanced BC Following CDK4/6 Inhibitor Therapy (EMERALD)	Interventional (Phase III trial)	Complete	Arm A: Elacestrant Arm B: Standard of care	ER-positive/HER2-BC	Metastatic	Outcome measure	478
NCT04964934	Switching to AZD9833 (a Next Generation, Oral SERD) + CDK4/6 Inhibitor vs Continuing Aromatase Inhibitor + CDK4/6 Inhibitor in HR+/HER2-MBC Patients With Detectable ESR1Mutation Without Disease Progression During 1L Treatment With Aromatase Inhibitor + CDK4/6 Inhibitor (SERENA-06)	Interventional (Phase III)	Complete	Arm A: AZD9833 + palbociclib, abemaciclib or ribociclib Arm B: Anastrozole or letrozole + palbociclib, abemaciclib or ribociclib	ER-positive/HER2-BC	Metastatic	Randomization criteria	315

Of note, a significant limitation of surveying clinical trial protocols on clinicaltrials.gov is the frequent lack of information regarding the techniques of choice for the biomarker assessment. While this may represent only a minor limitation in the context of CTCs, where the standard is the detection and enumeration of EpCAM + cells, many different approaches are usually employed in the assessment of ctDNA and should be described in more detail.

## 9 Conclusions

BC is a heterogeneous disease, and several molecular aspects with pivotal clinical impact are still not fully elucidated. Early detection remains one of the most effective strategies to improve BC patients’ outcomes. Tissue biopsy is considered the gold standard for diagnosing BC and performing molecular characterization, but it has several limitations. LB promises to bypass these limits by monitoring treatment response and providing insightful information about disease progression.

Additionally, its application in the management of BC patients may offer a new opportunity to improve the therapeutic approach. Indeed, LB could provide an attractive alternative to traditional molecular profiling approaches. To date, multiple platforms are available. However, each methodology has different strengths and drawbacks, challenging the introduction of LB into clinical practice. Thus, establishing clinical standards and harmonizing procedures are crucial to validate LB as a reliable test complementary to the current practice.

## Ethical approval/patient consent:

This article is a narrative review based exclusively on previously published data and does not involve any new studies with human participants or animals performed by the authors. Therefore, ethical approval and informed consent were not required.

## Declaration of competing interest

The authors declare that they have no known competing financial interests or personal relationships that could have appeared to influence the work reported in this paper.

## References

[1] Sung H., Ferlay J., Siegel R.L., Laversanne M., Soerjomataram I., Jemal A. (2021). Global cancer statistics 2020: GLOBOCAN estimates of incidence and mortality worldwide for 36 cancers in 185 countries. CA Cancer J Clin.

[2] Lafourcade A., His M., Baglietto L., Boutron-Ruault M.-C., Dossus L., Rondeau V. (2018). Factors associated with breast cancer recurrences or mortality and dynamic prediction of death using history of cancer recurrences: the French E3N cohort. BMC Cancer.

[3] Igari F., Tanaka H., Giuliano A.E. (2022). The applications of plasma cell-free DNA in cancer detection: implications in the management of breast cancer patients. Crit Rev Oncol Hematol.

[4] Martins I., Ribeiro I.P., Jorge J., Gonçalves A.C., Sarmento-Ribeiro A.B., Melo J.B. (2021). Liquid biopsies: applications for cancer diagnosis and monitoring. Genes.

[5] Kalinowski L., Saunus J.M., McCart Reed A.E., Lakhani S.R., Ahmad A. (2019).

[6] Fusco N., Ragazzi M., Sajjadi E., Venetis K., Piciotti R., Morganti S. (2021). Assessment of estrogen receptor low positive status in breast cancer: implications for pathologists and oncologists. Histol Histopathol.

[7] Nicolini A., Ferrari P., Duffy M.J. (2018). Prognostic and predictive biomarkers in breast cancer: past, present and future. Semin Cancer Biol.

[8] Fanelli G.N., Naccarato A.G., Scatena C. (2020). Recent advances in cancer plasticity: cellular mechanisms, surveillance strategies, and therapeutic optimization. Front Oncol.

[9] Nicolò E., Serafini M.S., Munoz-Arcos L., Pontolillo L., Molteni E., Bayou N. (2023). Real-time assessment of HER2 status in circulating tumor cells of breast cancer patients: methods of detection and clinical implications. J Liq Biopsy.

[10] Smit D.J., Pantel K. (2024). Circulating tumor cells as liquid biopsy markers in cancer patients. Mol Aspect Med.

[11] Scatena C., Fanelli G., Fanelli G.N., Menicagli M., Aretini P., Ortenzi V. (2021). New insights in the expression of stromal caveolin 1 in breast cancer spread to axillary lymph nodes. Sci Rep.

[12] Khatami F, Aghayan HR, Sanaei M, Heshmat R, Tavangar SM. The potential of circulating tumor cells in personalized management of breast cancer: a systematic review. Circulating Tumor Cells n.d.:19. 28282718

[13] Fanelli G.N., Scarpitta R., Cinacchi P., Fuochi B., Szumera-Ciećkiewicz A., De Ieso K. (2021). Immunohistochemistry for thymidine Kinase-1 (TK1): a potential tool for the prognostic stratification of breast cancer patients. J Clin Med.

[14] Naccarato A.G., Viacava P., Vignati S., Fanelli G., Bonadio A.G., Montruccoli G. (2000). Bio-morphological events in the development of the human female mammary gland from fetal age to puberty. Virchows Arch.

[15] Pinzani P., Scatena C., Salvianti F., Corsini E., Canu L., Poli G. (2013). Detection of circulating tumor cells in patients with adrenocortical carcinoma: a monocentric preliminary study. J Clin Endocrinol Metab.

[16] Mazzini C., Pinzani P., Salvianti F., Scatena C., Paglierani M., Ucci F. (2014). Circulating tumor cells detection and counting in uveal melanomas by a filtration-based method. Cancers (Basel).

[17] Pasqualetti F., Giampietro C., Montemurro N., Giannini N., Gadducci G., Orlandi P. (2022). Old and new systemic immune-inflammation indexes are associated with overall survival of glioblastoma patients treated with radio-chemotherapy. Genes (Basel).

[18] Lin D., Shen L., Luo M., Zhang K., Li J., Yang Q. (2021). Circulating tumor cells: biology and clinical significance. Signal Transduct Targeted Ther.

[19] Pasqualetti F., Montemurro N., Desideri I., Loi M., Giannini N., Gadducci G. (2022). Impact of recurrence pattern in patients undergoing a second surgery for recurrent glioblastoma. Acta Neurol Belg.

[20] Mavrommati I., Johnson F., Echeverria G.V., Natrajan R. (2021). Subclonal heterogeneity and evolution in breast cancer. Npj Breast Cancer.

[21] Francini E., Fanelli G.N., Pederzoli F., Spisak S., Minonne E., Raffo M. (2022). Circulating cell-free DNA in renal cell carcinoma: the new era of precision medicine. Cancers (Basel).

[22] Nuzzo P.V., Berchuck J.E., Korthauer K., Spisak S., Nassar A.H., Abou Alaiwi S. (2020). Detection of renal cell carcinoma using plasma and urine cell-free DNA methylomes. Nat Med.

[23] Indraccolo S., Lombardi G., Fassan M., Pasqualini L., Giunco S., Marcato R. (2019). Genetic, epigenetic, and immunologic profiling of MMR-deficient relapsed glioblastoma. Clin Cancer Res.

[24] Cucchiara F., Scarpitta R., Crucitta S., Scatena C., Arici R., Naccarato A.G. (2022). Diagnosis and treatment monitoring in breast cancer: how liquid biopsy can support patient management. Pharmacogenomics.

[25] Bedard P.L., Hansen A.R., Ratain M.J., Siu L.L. (2013). Tumour heterogeneity in the clinic. Nature.

[26] Smerage J.B., Barlow W.E., Hortobagyi G.N., Winer E.P., Leyland-Jones B., Srkalovic G. (2014). Circulating tumor cells and response to chemotherapy in metastatic breast cancer: swog S0500. J Clin Orthod.

[27] Sefrioui D., Blanchard F., Toure E., Basile P., Beaussire L., Dolfus C. (2017). Diagnostic value of CA19.9, circulating tumour DNA and circulating tumour cells in patients with solid pancreatic tumours. Br J Cancer.

[28] Rodríguez J., Avila J., Rolfo C., Ruíz-Patiño A., Russo A., Ricaurte L. (2021). When tissue is an issue the liquid biopsy is nonissue: a review. Oncol Ther.

[29] Venetis K., Cursano G., Pescia C., D'Ercole M., Porta F.M., Blanco M.C. (2023). Liquid biopsy: cell-Free DNA based analysis in breast cancer. J Liq Biopsy.

[30] Ranghiero A., Frascarelli C., Cursano G., Pescia C., Ivanova M., Vacirca D. (2023). Circulating tumour DNA testing in metastatic breast cancer: integration with tissue testing. Cytopathology.

[31] Arechederra M., Ávila M.A., Berasain C. (2020). Liquid biopsy for cancer management: a revolutionary but still limited new tool for precision medicine. Adv Lab Med/Avances En Medicina de Laboratorio.

[32] Lone S.N., Nisar S., Masoodi T., Singh M., Rizwan A., Hashem S. (2022). Liquid biopsy: a step closer to transform diagnosis, prognosis and future of cancer treatments. Mol Cancer.

[33] Wu J., Hu S., Zhang L., Xin J., Sun C., Wang L. (2020). Tumor circulome in the liquid biopsies for cancer diagnosis and prognosis. Theranostics.

[34] Lianidou E., Pantel K. (2019). Liquid biopsies. Genes Chromosomes Cancer.

[35] Yang Y., Zhang H., Zhang M., Meng Q., Cai L., Zhang Q. (2017). Elevation of serum CEA and CA15-3 levels during antitumor therapy predicts poor therapeutic response in advanced breast cancer patients. Oncol Lett.

[36] Duffy M.J., Crown J. (2022). Circulating tumor DNA as a biomarker for monitoring patients with solid cancers: Comparison with standard protein biomarkers. Clin Chem.

[37] Shao Y., Sun X., He Y., Liu C., Liu H. (2015). Elevated levels of serum tumor markers CEA and CA15-3 are prognostic parameters for different molecular subtypes of breast cancer. PLoS One.

[38] Hasan D. (2022). Diagnostic impact of CEA and CA 15-3 on monitoring chemotherapy of breast cancer patients: chemotherapy monitoring by serum markers. J Circ Biomark.

[39] Hall C., Clarke L., Pal A., Buchwald P., Eglinton T., Wakeman C. (2019). A review of the role of carcinoembryonic antigen in clinical practice. Ann Coloproctol.

[40] Anoop T.M., Joseph P.R., Soman S., Chacko S., Mathew M. (2022). Significance of serum carcinoembryonic antigen in metastatic breast cancer patients: a prospective study. WJCO.

[41] Uygur M.M., Gümüş M. (2021). The utility of serum tumor markers CEA and CA 15–3 for breast cancer prognosis and their association with clinicopathological parameters. Cancer Treat Res Comm.

[42] Lan Y., Ni W., Tai G. (2022). Expression of MUC1 in different tumours and its clinical significance. Mol Clin Oncol.

[43] Duffy M.J., McDermott E.W., Crown J. (2018). Blood-based biomarkers in breast cancer: from proteins to circulating tumor cells to circulating tumor DNA. Tumour Biol.

[44] Milosevic B., Stojanovic B., Cvetkovic A., Jovanovic I., Spasic M., Stojanovic M.D. (2023). The enigma of mammaglobin: redefining the biomarker paradigm in breast carcinoma. IJMS.

[45] Fatima M., Sai Baba K.S.S., Sreedevi N.N.R., Kumar J.P., Raju G.S., Uppin S.G. (2023). Evaluation of serum mammaglobin as an alternative biomarker in the diagnosis of breast tumors. J Lab Physicians.

[46] Gorbokon N., Timm P., Dum D., Menz A., Büscheck F., Völkel C. (2023). Mammaglobin-A expression is highly specific for tumors derived from the breast, the female genital tract, and the salivary gland. Diagnostics (Basel).

[47] Fatima M., Sai Baba K.S.S., Sreedevi N.N.R., Kumar J.P., Raju G.S., Uppin S.G. (2022). Evaluation of serum mammaglobin as an alternative biomarker in the diagnosis of breast tumors. J Lab Physicians.

[48] Monsalve-Lancheros A., Ibáñez-Pinilla M., Ramírez-Clavijo S. (2019). Detection of mammagloblin by RT-PCR as a biomarker for lymph node metastasis in breast cancer patients: a systematic review and meta-analysis. PLoS One.

[49] Fatima M., Sai Baba K.S.S., Sreedevi N.N.R., Kumar J.P., Raju G.S., Uppin S.G. (2023). Evaluation of serum mammaglobin as an alternative biomarker in the diagnosis of breast tumors. J Lab Physicians.

[50] Monsalve-Lancheros A., Ibáñez-Pinilla M., Ramírez-Clavijo S. (2019). Detection of mammagloblin by RT-PCR as a biomarker for lymph node metastasis in breast cancer patients: a systematic review and meta-analysis. PLoS One.

[51] Shang J., Zhao M., Deng H., Liu C., Cai L., Liu Y. (2022). A clinical diagnostic test on the detection of sentinel lymph node metastasis in breast neoplasms using a 1-step RT-PCR. Gland Surg.

[52] Wang L. (2017). Early diagnosis of breast cancer. Sensors.

[53] Addanki S., Meas S., Sarli V.N., Singh B., Lucci A. (2022). Applications of circulating tumor cells and circulating tumor DNA in precision oncology for breast cancers. IJMS.

[54] Addanki S., Meas S., Sarli V.N., Singh B., Lucci A. (2022). Applications of circulating tumor cells and circulating tumor DNA in precision oncology for breast cancers. IJMS.

[55] Tellez-Gabriel M., Knutsen E., Perander M. (2020). Current status of circulating tumor cells, circulating tumor DNA, and exosomes in breast cancer liquid biopsies. IJMS.

[56] Dasgupta A., Lim A.R., Ghajar C.M. (2017). Circulating and disseminated tumor cells: harbingers or initiators of metastasis?. Mol Oncol.

[57] Cristofanilli M., Stopeck A., Reuben J.M. (2004). Circulating tumor cells, disease progression, and survival in metastatic breast cancer. N Engl J Med.

[58] Thomas-Bonafos T., Pierga J.Y., Bidard F.-C., Cabel L., Kiavue N. (2024). Circulating tumor cells in breast cancer: clinical validity and utility. NPJ Breast Cancer.

[59] Cristofanilli M., Hayes D.F., Budd G.T., Ellis M.J., Stopeck A., Reuben J.M. (2005). Circulating tumor cells: a novel prognostic factor for newly diagnosed metastatic breast cancer. J Clin Orthod.

[60] D'Amico P., Corvaja C., Gerratana L., Reduzzi C., Curigliano G., Cristofanilli M. (2021). The use of liquid biopsy in early breast cancer: clinical evidence and future perspectives. JCMT.

[61] Brechbuhl H.M., Vinod-Paul K., Gillen A.E., Kopin E.G., Gibney K., Elias A.D. (2020). Analysis of circulating breast cancer cell heterogeneity and interactions with peripheral blood mononuclear cells. Mol Carcinog.

[62] Gu X., Wei S., Lv X. (2024). Circulating tumor cells: from new biological insights to clinical practice. Signal Transduct Targeted Ther.

[63] Lin D., Shen L., Luo M., Zhang K., Li J., Yang Q. (2021). Circulating tumor cells: biology and clinical significance. Signal Transduct Targeted Ther.

[64] Gu X., Wei S., Lv X. (2024). Circulating tumor cells: from new biological insights to clinical practice. Signal Transduct Targeted Ther.

[65] Guan X., Ma F., Li C., Wu S., Hu S., Huang J. (2019). The prognostic and therapeutic implications of circulating tumor cell phenotype detection based on epithelial-mesenchymal transition markers in the first-line chemotherapy of HER2-negative metastatic breast cancer. Cancer Commun.

[66] Zhang S., Wu T., Peng X., Liu J., Liu F., Wu S. (2017). Mesenchymal phenotype of circulating tumor cells is associated with distant metastasis in breast cancer patients. CMAR.

[67] Wang L., Balasubramanian P., Chen A.P., Kummar S., Evrard Y.A., Kinders R.J. (2016). Promise and limits of the CellSearch platform for evaluating pharmacodynamics in circulating tumor cells. Semin Oncol.

[68] Eslami S.Z., Cortés-Hernández L.E., Alix-Panabières C. (2020). Epithelial cell adhesion molecule: an anchor to isolate clinically relevant circulating tumor cells. Cells.

[69] Brown T.C., Sankpal N.V., Gillanders W.E. (2021). Functional implications of the dynamic regulation of EpCAM during epithelial-to-mesenchymal transition. Biomolecules.

[70] Pantel K., Speicher M.R. (2016). The biology of circulating tumor cells. Oncogene.

[71] Millner L.M., Linder M.W., Valdes R. (2016). Circulating tumor cells: a review of present methods and the need to identify heterogeneous phenotypes.

[72] Feng Z., Wu J., Lu Y., Chan Y.-T., Zhang C., Wang D. (2022). Circulating tumor cells in the early detection of human cancers. Int J Biol Sci.

[73] Hu X., Zang X., Lv Y. (2021). Detection of circulating tumor cells: advances and critical concerns. Oncol Lett.

[74] Bahadoran E., Moghbelinejad S., Mohammadi G., Shahbazmohammadi H., Abdolvahabi Z., Jalilvand M. (2025). Cytokeratin expression in breast cancer: from mechanisms, progression, diagnosis, and prognosis to therapeutic implications. Mol Cell Oncol.

[75] Safadi R.A., Abdullah N.I., Alaaraj R.F., Bader D.H., Divakar D.D., Hamasha A.A. (2019). Clinical and histopathologic prognostic implications of the expression of cytokeratins 8, 10, 13, 14, 16, 18 and 19 in oral and oropharyngeal squamous cell carcinoma. Arch Oral Biol.

[76] Keyvani S., Karimi N., Orafa Z., Bouzari S., Oloomi M. (2016). Assessment of Cytokeratin-19 gene expression in peripheral blood of breast cancer patients and breast cancer cell lines. Biomark Cancer.

[77] Menz A., Bauer R., Kluth M., Marie von Bargen C., Gorbokon N., Viehweger F. (2021). Diagnostic and prognostic impact of cytokeratin 19 expression analysis in human tumors: a tissue microarray study of 13,172 tumors. Hum Pathol.

[78] Matikas A., Kotsakis A., Apostolaki S., Politaki H., Perraki M., Kalbakis K. (2022). Detection of circulating tumour cells before and following adjuvant chemotherapy and long-term prognosis of early breast cancer. Br J Cancer.

[79] Jie X.-X., Zhang X.-Y., Xu C.-J. (2017). Epithelial-to-mesenchymal transition, circulating tumor cells and cancer metastasis: mechanisms and clinical applications. Oncotarget.

[80] Tayoun Faugeroux, Oulhen Aberlenc, Pawlikowska Farace (2019). CTC-derived models: a window into the seeding capacity of circulating tumor cells (CTCs). Cells.

[81] Agnoletto C., Corrà F., Minotti L., Baldassari F., Crudele F., Cook W. (2019). Heterogeneity in circulating tumor cells: the relevance of the stem-cell subset. Cancers.

[82] Yu T., Wang C., Xie M., Zhu C., Shu Y., Tang J. (2021). Heterogeneity of CTC contributes to the organotropism of breast cancer. Biomed Pharmacother.

[83] Menyailo M.E., Tretyakova M.S., Denisov E.V. (2020). Heterogeneity of circulating tumor cells in breast cancer: identifying metastatic seeds. IJMS.

[84] Bronkhorst A.J., Ungerer V., Holdenrieder S. (2019). The emerging role of cell-free DNA as a molecular marker for cancer management. Biomol Detection Quantification.

[85] Underhill H.R., Kitzman J.O., Hellwig S., Welker N.C., Daza R., Baker D.N. (2016). Fragment length of circulating tumor DNA. PLoS Genet.

[86] Bredno J., Lipson J., Venn O., Aravanis A.M., Jamshidi A. (2021). Clinical correlates of circulating cell-free DNA tumor fraction. PLoS One.

[87] Sausen M., Parpart S., Diaz L.A. (2014). Circulating tumor DNA moves further into the spotlight. Genome Med.

[88] Keller L., Belloum Y., Wikman H., Pantel K. (2021). Clinical relevance of blood-based ctDNA analysis: mutation detection and beyond. Br J Cancer.

[89] Alborelli I., Generali D., Jermann P., Cappelletti M.R., Ferrero G., Scaggiante B. (2019). Cell-free DNA analysis in healthy individuals by next-generation sequencing: a proof of concept and technical validation study. Cell Death Dis.

[90] Corcoran R.B., Chabner B.A. (2018). Application of cell-free DNA analysis to cancer treatment. N Engl J Med.

[91] Yan Y.-Y., Guo Q.-R., Wang F.-H., Adhikari R., Zhu Z.-Y., Zhang H.-Y. (2021). Cell-free DNA: hope and potential application in cancer. Front Cell Dev Biol.

[92] Guo Q., Hua Y. (2021). The assessment of circulating cell-free DNA as a diagnostic tool for breast cancer: an updated systematic review and meta-analysis of quantitative and qualitative ssays. Clin Chem Lab Med.

[93] Diaz L.A., Bardelli A. (2014). Liquid biopsies: genotyping circulating tumor DNA. J Clin Orthod.

[94] Tamborero D., Rubio-Perez C., Deu-Pons J., Schroeder M.P., Vivancos A., Rovira A. (2018). Cancer genome interpreter annotates the biological and clinical relevance of tumor alterations. Genome Med.

[95] Hasenleithner S.O., Speicher M.R. (2022). A clinician's handbook for using ctDNA throughout the patient journey. Mol Cancer.

[96] Tivey A., Church M., Rothwell D., Dive C., Cook N. (2022). Circulating tumour DNA — looking beyond the blood. Nat Rev Clin Oncol.

[97] Keller L., Belloum Y., Wikman H., Pantel K. (2021). Clinical relevance of blood-based ctDNA analysis: mutation detection and beyond. Br J Cancer.

[98] Heidrich I., Deitert B., Werner S., Pantel K. (2023). Liquid biopsy for monitoring of tumor dormancy and early detection of disease recurrence in solid tumors. Cancer Metastasis Rev.

[99] Malik S., Zaheer S. (2025). The impact of liquid biopsy in breast cancer: redefining the landscape of non-invasive precision oncology. J Liq Biopsy.

[100] Stadler J.-C., Belloum Y., Deitert B., Sementsov M., Heidrich I., Gebhardt C. (2022). Current and future clinical applications of ctDNA in immuno-oncology. Cancer Res.

[101] Shan N.L., Gould B., Wang X., Bonora G., Blenman K., Foldi J. (2024). Circulating tumor DNA fraction predicts residual cancer burden post-neoadjuvant chemotherapy in triple negative breast cancer. J Liq Biopsy.

[102] Tamkovich S., Tupikin A., Kozyakov A., Laktionov P. (2022). Size and methylation index of cell-free and cell-surface-bound DNA in blood of breast cancer patients in the contest of liquid biopsy. IJMS.

[103] Tamkovich S., Laktionov P. (2019). Cell‐surface‐bound circulating DNA in the blood biology and clinical application.

[104] Afzal S., Hassan M., Ullah S., Abbas H., Tawakkal F., Khan M.A. (2022). Breast cancer; discovery of novel diagnostic biomarkers, drug resistance, and therapeutic implications. Front Mol Biosci.

[105] Andree K.C., van Dalum G., Terstappen L.W.M.M. (2016). Challenges in circulating tumor cell detection by the CellSearch system. Mol Oncol.

[106] Thery L., Meddis A., Cabel L., Proudhon C., Latouche A., Pierga J.-Y. (2019). Circulating tumor cells in early breast cancer. JNCI Cancer Spectr.

[107] Bidard F.-C., Michiels S., Riethdorf S., Mueller V., Esserman L.J., Lucci A. (2018). Circulating tumor cells in breast cancer patients treated by neoadjuvant chemotherapy: a meta-analysis. JNCI: J Natl Cancer Inst.

[108] Fabisiewicz A., Szostakowska-Rodzos M., Zaczek A.J., Grzybowska E.A. (2020). Circulating tumor cells in early and advanced breast cancer; biology and prognostic value. IJMS.

[109] Sant M., Bernat-Peguera A., Felip E., Margelí M. (2022). Role of ctDNA in breast cancer. Cancers.

[110] Martens G.A., Demol J., Dedeurwaerdere F., Breyne J., De Smet K., De Jaeger P. (2024). Rational thresholding of circulating tumor DNA concentration for improved surveillance of metastatic breast cancer. ESMO Open.

[111] Rohanizadegan M. (2018). Analysis of circulating tumor DNA in breast cancer as a diagnostic and prognostic biomarker. Cancer Genet.

[112] Lin Z., Neiswender J., Fang B., Ma X., Zhang J., Hu X. (2017). Value of circulating cell-free DNA analysis as a diagnostic tool for breast cancer: a meta-analysis. Oncotarget.

[113] Zill O.A., Banks K.C., Fairclough S.R., Mortimer S.A., Vowles J.V., Mokhtari R. (2018). The landscape of actionable genomic alterations in cell-free circulating tumor DNA from 21,807 advanced cancer patients. Clin Cancer Res.

[114] Grisolia P., Tufano R., Iannarone C., De Falco A., Carlino F., Graziano C. (2024). Differential methylation of circulating free DNA assessed through cfMeDiP as a new tool for breast cancer diagnosis and detection of BRCA1/2 mutation. J Transl Med.

[115] Palanca-Ballester C., Rodriguez-Casanova A., Torres S., Calabuig-Fariñas S., Exposito F., Serrano D. (2021). Cancer epigenetic biomarkers in liquid biopsy for high incidence malignancies. Cancers.

[116] Mousavi-kiasary S.M.S., Bayat M., Abbasvandi F., Khoundabi B., Mousavi F., Akbari A. (2025). Tumor characteristics and survival rate of axillary metastatic breast cancer patients: a three decades retrospective cohort study. Sci Rep.

[117] Akrami M., Meshksar A., Ghoddusi J.M., Safarpour M.M., Tahmasebi S., Zangouri V. (2021). Prognostic role of lymphovascular invasion in patients with early breast cancer. Indian J Surg Oncol.

[118] Wang J., Wu S.-G. (2023). Breast cancer: an overview of current therapeutic strategies, challenge, and perspectives. Breast Cancer (Dove Med Press).

[119] Raimondi C., Nicolazzo C., Gradilone A. (2015). Circulating tumor cells isolation: the “post-EpCAM era.”. Chin J Cancer Res.

[120] Tirada N., Aujero M., Khorjekar G., Richards S., Chopra J., Dromi S. (2018). Breast cancer tissue markers, genomic profiling, and other prognostic factors: a primer for radiologists. Radiographics.

[121] Afzal S., Hassan M., Ullah S., Abbas H., Tawakkal F., Khan M.A. (2022). Breast cancer; discovery of novel diagnostic biomarkers, drug resistance, and therapeutic implications. Front Mol Biosci.

[122] Höller A., Nguyen-Sträuli B.D., Frauchiger-Heuer H., Ring A. (2023). Diagnostic and prognostic biomarkers of luminal breast cancer: where are we now?. Breast Cancer (Dove Med Press).

[123] Banys-Paluchowski M., Krawczyk N., Fehm T. (2016). Potential role of circulating tumor cell detection and monitoring in breast cancer: a review of current evidence. Front Oncol.

[124] Banys-Paluchowski M., Krawczyk N., Fehm T. (2016). Potential role of circulating tumor cell detection and monitoring in breast cancer: a review of current evidence. Front Oncol.

[125] Jongbloed E.M., Deger T., Sleijfer S., Martens J.W.M., Jager A., Wilting S.M. (2021). A systematic review of the use of circulating cell-free DNA dynamics to monitor response to treatment in metastatic breast cancer patients. Cancers (Basel).

[126] Bilani N., Elson L., Liang H., Elimimian E.B., Arteta-Bulos R., Nahleh Z. (2020). Prognostic and predictive value of circulating and disseminated tumor cells in breast cancer: a national cancer database (NCDB) analysis. Technol Cancer Res Treat.

[127] Bahnassy A.A., Saber M.M., Mahmoud M.G., Abdellateif M.S., Abd El-Mooti Samra M., Abd El-Fatah R.M. (2018). The role of circulating tumor cells in metastatic breast cancer: prognostic and predictive value. Mol Biol Rep.

[128] Bidard F.-C., Jacot W., Kiavue N., Dureau S., Kadi A., Brain E. (2021). Efficacy of circulating tumor cell count-driven vs clinician-driven first-line therapy choice in hormone receptor-positive, ERBB2-Negative metastatic breast cancer: the STIC CTC randomized clinical trial. JAMA Oncol.

[129] Cristofanilli M., Pierga J.-Y., Reuben J., Rademaker A., Davis A.A., Peeters D.J. (2019). The clinical use of circulating tumor cells (CTCs) enumeration for staging of metastatic breast cancer (MBC): international expert consensus paper. Crit Rev Oncol Hematol.

[130] Luo M., Brooks M., Wicha M. (2015). Epithelial-mesenchymal plasticity of breast cancer stem cells: implications for metastasis and therapeutic resistance. CPD.

[131] Lawson D.A., Bhakta N.R., Kessenbrock K., Prummel K.D., Yu Y., Takai K. (2015). Single-cell analysis reveals a stem-cell program in human metastatic breast cancer cells. Nature.

[132] Gonzalez H., Hagerling C., Werb Z. (2018). Roles of the immune system in cancer: from tumor initiation to metastatic progression. Genes Dev.

[133] Pang S., Li H., Xu S., Feng L., Ma X., Chu Y. (2021). Circulating tumour cells at baseline and late phase of treatment provide prognostic value in breast cancer. Sci Rep.

[134] Ring A., Spataro M., Wicki A., Aceto N. (2022). Clinical and biological aspects of disseminated tumor cells and dormancy in breast cancer. Front Cell Dev Biol.

[135] Masuda T., Hayashi N., Iguchi T., Ito S., Eguchi H., Mimori K. (2016). Clinical and biological significance of circulating tumor cells in cancer. Mol Oncol.

[136] Janni W., Friedl T.W.P., Yab T.C., Bidard F.-C., Cristofanilli M., Hayes D.F. (2025). Clinical validity of repeated circulating tumor cell enumeration as an early treatment monitoring tool for metastatic breast cancer in the PREDICT global pooled analysis. Clin Cancer Res.

[137] Broersen L.H.A., van Pelt G.W., Tollenaar R.A.E.M., Mesker W.E. (2014). Clinical application of circulating tumor cells in breast cancer. Cell Oncol (Dordr).

[138] Matikas A., Kotsakis A., Apostolaki S., Politaki H., Perraki M., Kalbakis K. (2022). Detection of circulating tumour cells before and following adjuvant chemotherapy and long-term prognosis of early breast cancer. Br J Cancer.

[139] Fridrichova I., Kalinkova L., Ciernikova S. (2022). Clinical relevancy of circulating tumor cells in breast cancer: epithelial or mesenchymal characteristics, single cells or clusters?. IJMS.

[140] Mego M., Giordano A., De Giorgi U., Masuda H., Hsu L., Giuliano M. (2015). Circulating tumor cells in newly diagnosed inflammatory breast cancer. Breast Cancer Res.

[141] Lv Q., Gong L., Zhang T., Ye J., Chai L., Ni C. (2016). Prognostic value of circulating tumor cells in metastatic breast cancer: a systemic review and meta-analysis. Clin Transl Oncol.

[142] Lu Y., Wang P., Wang X., Peng J., Zhu Y., Shen N. (2016). The significant prognostic value of circulating tumor cells in triple-negative breast cancer: a meta-analysis. Oncotarget.

[143] Dhaka S., Tripathi R., Doval D.C., Mehta A., Maheshwari U., Koyyala V.P.B. (2023). Role of circulating tumor cells in determining prognosis in metastatic breast cancer. South Asian J Cancer.

[144] Lisencu L.A., Trancă S., Bonci E.-A., Paşca A., Mihu C., Irimie A. (2022). The role of circulating tumor cells in the prognosis of metastatic triple-negative breast cancers: a systematic review of the literature. Biomedicines.

[145] Lu Y.-J., Wang P., Wang X., Peng J., Zhu Y.-W., Shen N. (2016). The significant prognostic value of circulating tumor cells in triple-negative breast cancer: a meta-analysis. Oncotarget.

[146] Mego M., Giordano A., De Giorgi U., Masuda H., Hsu L., Giuliano M. (2015). Circulating tumor cells in newly diagnosed inflammatory breast cancer. Breast Cancer Res.

[147] Lozar T., Gersak K., Cemazar M., Kuhar C.G., Jesenko T. (2019). The biology and clinical potential of circulating tumor cells. Radiol Oncol.

[148] Xiao J., Pohlmann P.R., Isaacs C., Weinberg B.A., He A.R., Schlegel R. (2021). Circulating tumor cells: technologies and their clinical potential in cancer metastasis. Biomedicines.

[149] Tabor S., Szostakowska-Rodzos M., Fabisiewicz A., Grzybowska E.A. (2020). How to predict metastasis in luminal breast cancer? Current solutions and future prospects. IJMS.

[150] Scatena C., Roncella M., Di Paolo A., Aretini P., Menicagli M., Fanelli G. (2018). Doxycycline, an inhibitor of mitochondrial biogenesis, effectively reduces cancer stem cells (CSCs) in early breast cancer patients: a clinical pilot study. Front Oncol.

[151] Gomatou G., Syrigos N., Vathiotis I.A., Kotteas E.A. (2021). Tumor dormancy: implications for invasion and metastasis. Int J Mol Sci.

[152] Tachtsidis A., McInnes L.M., Jacobsen N., Thompson E.W., Saunders C.M. (2016). Minimal residual disease in breast cancer: an overview of circulating and disseminated tumour cells. Clin Exp Metastasis.

[153] Lin S.Y., Orozco J.I.J., Hoon D.S.B., Aguirre-Ghiso J.A. (2018).

[154] Alemzadeh E., Allahqoli L., Dehghan H., Mazidimoradi A., Ghasempour A., Salehiniya H. (2023). Circulating tumor cells and circulating tumor DNA in breast cancer diagnosis and monitoring. Oncol Res.

[155] Lambert A.W., Pattabiraman D.R., Weinberg R.A. (2017). Emerging biological principles of metastasis. Cell.

[156] Fabricius H.-Å., Starzonek S., Lange T. (2021). The role of platelet cell surface P-Selectin for the direct platelet-tumor cell contact during metastasis formation in human tumors. Front Oncol.

[157] Lu Y.-J., Cui M.-T., Liang Z.-W., Wang W.-J., Jiang M., Xu M.-D. (2019). Prognostic values of platelet-associated indicators in advanced breast cancer. Transl Cancer Res.

[158] Freitas AJA de, Causin R.L., Varuzza M.B., Calfa S., Hidalgo Filho C.M.T., Komoto T.T. (2022). Liquid biopsy as a tool for the diagnosis, treatment, and monitoring of breast cancer. IJMS.

[159] Peng Y., Mei W., Ma K., Zeng C. (2021). Circulating tumor DNA and minimal residual disease (MRD) in solid tumors: Current Horizons and future perspectives. Front Oncol.

[160] Ferrari P., Scatena C., Ghilli M., Bargagna I., Lorenzini G., Nicolini A. (2022). Molecular mechanisms, biomarkers and emerging therapies for chemotherapy resistant TNBC. Int J Mol Sci.

[161] Chen Y.-H., Hancock B.A., Solzak J.P., Brinza D., Scafe C., Miller K.D. (2017). Next-generation sequencing of circulating tumor DNA to predict recurrence in triple-negative breast cancer patients with residual disease after neoadjuvant chemotherapy. Npj Breast Cancer.

[162] Riva F., Bidard F.-C., Houy A., Saliou A., Madic J., Rampanou A. (2017). Patient-specific circulating tumor DNA detection during neoadjuvant chemotherapy in triple-negative breast cancer. Clin Chem.

[163] Madic J., Kiialainen A., Bidard F.-C., Birzele F., Ramey G., Leroy Q. (2015). Circulating tumor DNA and circulating tumor cells in metastatic triple negative breast cancer patients: ctDNA and CTC in metastatic triple negative breast cancer. Int J Cancer.

[164] Visvanathan K., Fackler M.S., Zhang Z., Lopez-Bujanda Z.A., Jeter S.C., Sokoll L.J. (2017). Monitoring of serum DNA methylation as an early independent marker of response and survival in metastatic breast cancer: TBCRC 005 prospective biomarker study. J Clin Oncol.

[165] Oshiro C., Kagara N., Naoi Y., Shimoda M., Shimomura A., Maruyama N. (2015). PIK3CA mutations in serum DNA are predictive of recurrence in primary breast cancer patients. Breast Cancer Res Treat.

[166] Li L., Sun Y. (2024). Circulating tumor DNA methylation detection as biomarker and its application in tumor liquid biopsy: advances and challenges. MedComm.

[167] Zarean E., Li S., Southey M.C., Dugué P.-A. (2025). A review of the use of tumour DNA methylation for breast cancer subtyping and prediction of outcomes. Clin Epigenet.

[168] Panagopoulou M., Karaglani M., Balgkouranidou I., Biziota E., Koukaki T., Karamitrousis E. (2019). Circulating cell-free DNA in breast cancer: size profiling, levels, and methylation patterns lead to prognostic and predictive classifiers. Oncogene.

[169] Brooks J., Cairns P., Zeleniuch-Jacquotte A. (2009). Promoter methylation and the detection of breast cancer. Cancer Causes Control.

[170] Tanić M., Beck S. (2017). Epigenome-wide association studies for cancer biomarker discovery in circulating cell-free DNA: technical advances and challenges. Curr Opin Genet Dev.

[171] Warton K., Mahon K.L., Samimi G. (2016). Methylated circulating tumor DNA in blood: power in cancer prognosis and response. Endocrine-Related Cancer.

[172] Capp J.-P. (2021). Interplay between genetic, epigenetic, and gene expression variability: considering complexity in evolvability. Evol Appl.

[173] Esain-Garcia I. (2023). Deciphering the cancer genome and epigenome. Nat Rev Cancer.

[174] Holčáková J. (2018). Effect of DNA methylation on the development of cancer. Klin Onkol.

[175] Baylin S.B., Jones P.A. (2016). Epigenetic determinants of cancer. Cold Spring Harbor Perspect Biol.

[176] Casalino L., Verde P. (2020). Multifaceted roles of DNA methylation in neoplastic transformation, from tumor suppressors to EMT and metastasis. Genes (Basel).

[177] Connolly R.M., Fackler M.J., Zhang Z., Zhou X.C., Goetz M.P., Boughey J.C. (2018). Tumor and serum DNA methylation in women receiving preoperative chemotherapy with or without vorinostat in TBCRC008. Breast Cancer Res Treat.

[178] Nunes S.P., Moreira-Barbosa C., Salta S., Palma de Sousa S., Pousa I., Oliveira J. (2018). Cell-free DNA methylation of selected genes allows for early detection of the major cancers in women. Cancers (Basel).

[179] Fang C., Wei X.-M., Zeng X.-T., Wang F.-B., Weng H., Long X. (2015). Aberrant GSTP1 promoter methylation is associated with increased risk and advanced stage of breast cancer: a meta-analysis of 19 case-control studies. BMC Cancer.

[180] Kristiansen S., Nielsen D., Sölétormos G. (2016). Detection and monitoring of hypermethylated RASSF1A in serum from patients with metastatic breast cancer. Clin Epigenet.

[181] Swellam M., Abdelmaksoud M.D.E., Sayed Mahmoud M., Ramadan A., Abdel-Moneem W., Hefny M.M. (2015). Aberrant methylation of APC and RARβ2 genes in breast cancer patients. IUBMB Life.

[182] Shao Y., Sun X., He Y., Liu C., Liu H. (2015). Elevated levels of serum tumor markers CEA and CA15-3 are prognostic parameters for different molecular subtypes of breast cancer. PLoS One.

[183] Li M., Wang C., Yu B., Zhang X., Shi F., Liu X. (2019). Diagnostic value of RASSF1A methylation for breast cancer: a meta-analysis. Biosci Rep.

[184] Gupta V., Agarwal P., Deshpande P. (2021). Impact of RASSF1A gene methylation on clinico-pathological features of tumor and non-tumor tissue of breast cancer. Ann Diagn Pathol.

[185] Zhou D., Tang W., Wang W., Pan X., An H.-X., Zhang Y. (2016). Association between aberrant APC promoter methylation and breast cancer pathogenesis: a meta-analysis of 35 observational studies. PeerJ.

[186] He K., Zhang L., Long X. (2016). Quantitative assessment of the association between APC promoter methylation and breast cancer. Oncotarget.

[187] Saelee P., Pongtheerat T. (2020). APC promoter hypermethylation as a prognostic marker in breast cancer patients. Asian Pac J Cancer Prev.

[188] Swellam M., Abdelmaksoud M.D.E., Sayed Mahmoud M., Ramadan A., Abdel-Moneem W., Hefny M.M. (2015). Aberrant methylation of APC and RARβ2 genes in breast cancer patients. IUBMB Life.

[189] Warton K., Mahon K.L., Samimi G. (2016). Methylated circulating tumor DNA in blood: power in cancer prognosis and response. Endocr Relat Cancer.

[190] Ravera F., Dameri M., Nuzzo P.V., Stabile M., Fregatti P., Ballestrero A. (2024). Comprehensive analysis of plasma methylome reveals distinct patterns of methylation changes between responders and non-responders to neoadjuvant chemotherapy in breast cancer. J Liq Biopsy.

[191] Takahashi H., Kagara N., Tanei T., Naoi Y., Shimoda M., Shimomura A. (2017). Correlation of methylated circulating tumor DNA with response to neoadjuvant chemotherapy in breast cancer patients. Clin Breast Cancer.

[192] Janssen L.M., Janse M.H.A., Penning de Vries B.B.L., van der Velden B.H.M., Wolters-van der Ben E.J.M., van den Bosch S.M. (2024). Predicting response to neoadjuvant chemotherapy with liquid biopsies and multiparametric MRI in patients with breast cancer. NPJ Breast Cancer.

[193] Bahnassy A.A., Saber M.M., Mahmoud M.G., Abdellateif M.S., Abd El-Mooti Samra M., Abd El-Fatah R.M. (2018). The role of circulating tumor cells in metastatic breast cancer: prognostic and predictive value. Mol Biol Rep.

[194] Ye Z., Wang C., Wan S., Mu Z., Zhang Z., Abu-Khalaf M.M. (2019). Association of clinical outcomes in metastatic breast cancer patients with circulating tumour cell and circulating cell-free DNA. Eur J Cancer.

[195] Riethdorf S., Müller V., Loibl S., Nekljudova V., Weber K., Huober J. (2017). Prognostic impact of circulating tumor cells for breast cancer patients treated in the neoadjuvant “geparquattro” trial. Clin Cancer Res.

[196] Magbanua M.J.M., Hendrix L.H., Hyslop T., Barry W.T., Winer E.P., Hudis C. (2021). Serial analysis of circulating tumor cells in metastatic breast cancer receiving first-line chemotherapy. J Natl Cancer Inst.

[197] Hayes D.F., Cristofanilli M., Budd G.T., Ellis M.J., Stopeck A., Miller M.C. (2006). Circulating tumor cells at each Follow-up time point during therapy of metastatic breast cancer patients predict progression-free and overall survival. Clin Cancer Res.

[198] Deutsch T.M., Riethdorf S., Fremd C., Feisst M., Nees J., Fischer C. (2020). HER2-targeted therapy influences CTC status in metastatic breast cancer. Breast Cancer Res Treat.

[199] Müller V., Banys-Paluchowski M., Friedl T.W.P., Fasching P.A., Schneeweiss A., Hartkopf A. (2021). Prognostic relevance of the HER2 status of circulating tumor cells in metastatic breast cancer patients screened for participation in the DETECT study program. ESMO Open.

[200] Beije N., Onstenk W., Kraan J., Sieuwerts A.M., Hamberg P., Dirix L.Y. (2016). Prognostic impact of HER2 and ER status of circulating tumor cells in metastatic breast cancer patients with a HER2-Negative primary tumor. Neoplasia.

[201] Jordan N.V., Bardia A., Wittner B.S., Benes C., Ligorio M., Zheng Y. (2016). HER2 expression identifies dynamic functional states within circulating breast cancer cells. Nature.

[202] Volmer L.L., Dannehl D., Matovina S., Taran F.-A., Walter C.B., Wallwiener M. (2024). Comparing the HER2 status of the primary tumor to that of disseminated tumor cells in early breast cancer. Int J Mol Sci.

[203] Aaltonen K.E., Novosadová V., Bendahl P.-O., Graffman C., Larsson A.-M., Rydén L. (2017). Molecular characterization of circulating tumor cells from patients with metastatic breast cancer reflects evolutionary changes in gene expression under the pressure of systemic therapy. Oncotarget.

[204] Onstenk W., Sieuwerts A.M., Weekhout M., Mostert B., Reijm E.A., van Deurzen C.H.M. (2015). Gene expression profiles of circulating tumor cells versus primary tumors in metastatic breast cancer. Cancer Lett.

[205] Kalinsky K., Mayer J.A., Xu X., Pham T., Wong K.L., Villarin E. (2015). Correlation of hormone receptor status between circulating tumor cells, primary tumor, and metastasis in breast cancer patients. Clin Transl Oncol.

[206] Zuo W.-J., He M., Zheng H., Liu Y., Liu X.-Y., Jiang Y.-Z. (2021). Serum HER2 levels predict treatment efficacy and prognosis in patients with HER2-positive breast cancer undergoing neoadjuvant treatment. Gland Surg.

[207] Gheni N., Westenberg D. (2020). Quantitative real-time PCR assay with immunohistochemical evaluation of HER2/neu oncogene in breast cancer patients and its correlation with clinicopathological findings. Indian J Pathol Microbiol.

[208] Ring A., Spataro M., Wicki A., Aceto N. (2022). Clinical and biological aspects of disseminated tumor cells and dormancy in breast cancer. Front Cell Dev Biol.

[209] Hartkopf A.D., Wallwiener M., Hahn M., Fehm T.N., Brucker S.Y., Taran F.-A. (2016). Simultaneous detection of disseminated and circulating tumor cells in primary breast cancer patients. Cancer Res Treat.

[210] Walter V.P., Taran F.-A., Wallwiener M., Hahn M., Brucker S.Y., Hartkopf A.D. (2018). Simultaneous detection of circulating and disseminated tumor cells in primary breast cancer patients following neoadjuvant chemotherapy. Arch Gynecol Obstet.

[211] Rack B., Zombirt E., Trapp E., Jückstock J., Andergassen U., Neugebauer J. (2016). Comparison of HER2 expression in primary tumor and disseminated tumor cells in the bone marrow of breast cancer patients. Oncology.

[212] Gerratana L., Gianni C., Nicolò E., Pontolillo L., Bidard F.-C., Reduzzi C. (2025). Mapping breast cancer therapy with circulating tumor cells: the expert perspective. Breast.

[213] Shao B., Li H., Zhang J., Liu X., Song G., Jiang H. (2022). The prognostic value of chemotherapy or endocrine therapy choice according to circulating tumor cell count in HR+HER2− metastatic breast cancer: a retrospective study. Ann Transl Med.

[214] Bidard F.-C., Jacot W., Kiavue N., Dureau S., Kadi A., Brain E. (2021). Efficacy of circulating tumor cell count-driven vs clinician-driven first-line therapy choice in hormone receptor-positive, ERBB2-Negative metastatic breast cancer: the STIC CTC randomized clinical trial. JAMA Oncol.

[215] Gerratana L., Ren Y., Reduzzi C., Regan M.M., Mahtani R.L., Ma C.X. (2023). Circulating tumor cells (CTCs) dynamics after CDK4/6i for hormone-receptor positive (HR+) metastatic breast cancer (MBC): a biomarker analysis of the PACE randomized phase II study. J Clin Orthod.

[216] Fehm T., Mueller V., Banys-Paluchowski M., Fasching P.A., Friedl T.W.P., Hartkopf A. (2024). Efficacy of lapatinib in patients with HER2-Negative metastatic breast cancer and HER2-Positive circulating tumor cells-the DETECT III clinical trial. Clin Chem.

[217] Maltoni R., Palleschi M., Ravaioli S., Tumedei M.M., Rocca A., Melegari E. (2020). Cell-free DNA variant sequencing using CTC-depleted blood for comprehensive liquid biopsy testing in metastatic breast cancer. Cell Transplant.

[218] Olsson E., Winter C., George A., Chen Y., Howlin J., Tang M.E. (2015). Serial monitoring of circulating tumor DNA in patients with primary breast cancer for detection of occult metastatic disease. EMBO Mol Med.

[219] Moschetti I., Cinquini M., Lambertini M., Levaggi A., Liberati A. (2016). Follow-up strategies for women treated for early breast cancer. Cochrane Database Syst Rev.

[220] Carbine N.E., Lostumbo L., Wallace J., Ko H. (2018). Risk-reducing mastectomy for the prevention of primary breast cancer. Cochrane Database Syst Rev.

[221] Parsons H.A., Rhoades J., Reed S.C., Gydush G., Ram P., Exman P. (2020). Sensitive detection of minimal residual disease in patients treated for early-stage breast cancer. Clin Cancer Res.

[222] Pantel K., Alix-Panabières C. (2019). Liquid biopsy and minimal residual disease — latest advances and implications for cure. Nat Rev Clin Oncol.

[223] Scarpitta R., Zanna I., Aretini P., Gambino G., Scatena C., Mei B. (2019). Germline investigation in Male breast cancer of DNA repair genes by next-generation sequencing. Breast Cancer Res Treat.

[224] Pascual J., Attard G., Bidard F.-C., Curigliano G., De Mattos-Arruda L., Diehn M. (2022). ESMO recommendations on the use of circulating tumour DNA assays for patients with cancer: a report from the ESMO precision medicine working group. Ann Oncol.

[225] Rodriguez Córdoba, Aranda Álvarez, Vicioso Pérez (2019). Detection of TP53 and PIK3CA mutations in circulating tumor DNA using next-generation sequencing in the screening process for early breast cancer diagnosis. JCM.

[226] Saha S., Araf Y., Promon S.K. (2022). Circulating tumor DNA in cancer diagnosis, monitoring, and prognosis. J Egypt Natl Canc Inst.

[227] Wang R., Li X., Zhang H., Wang K., He J. (2017). Cell-free circulating tumor DNA analysis for breast cancer and its clinical utilization as a biomarker. Oncotarget.

[228] Sørensen P.D., Andersen R.F., Pallisgaard N., Madsen J.S., Jakobsen E.H., Brandslund I. (2015). Quantification of cell-free HER-2 DNA in plasma from breast cancer patients: sensitivity for detection of metastatic recurrence and gene amplification. J Circ Biomark.

[229] Page K., Hava N., Ward B., Brown J., Guttery D.S., Ruangpratheep C. (2011). Detection of HER2 amplification in circulating free DNA in patients with breast cancer. Br J Cancer.

[230] Chan J.C.H., Chow J.C.H., Ho C.H.M., Tsui T.Y.M., Cho W.C. (2021). Clinical application of circulating tumor DNA in breast cancer. J Cancer Res Clin Oncol.

[231] Engel C., Rhiem K., Hahnen E., Loibl S., Weber K.E., Seiler S. (2018). Prevalence of pathogenic BRCA1/2 germline mutations among 802 women with unilateral triple-negative breast cancer without family cancer history. BMC Cancer.

[232] Wong-Brown M.W., Meldrum C.J., Carpenter J.E., Clarke C.L., Narod S.A., Jakubowska A. (2015). Prevalence of BRCA1 and BRCA2 germline mutations in patients with triple-negative breast cancer. Breast Cancer Res Treat.

[233] Schreiber V., Illuzzi G., Héberlé E., Dantzer F. (2015). [from poly(ADP-ribose) discovery to PARP inhibitors in cancer therapy]. Bull Cancer.

[234] Robson M., Im S.-A., Senkus E., Xu B., Domchek S.M., Masuda N. (2017). Olaparib for metastatic breast cancer in patients with a germline *BRCA* mutation. N Engl J Med.

[235] Litton J.K., Rugo H.S., Ettl J., Hurvitz S.A., Gonçalves A., Lee K.-H. (2018). Talazoparib in patients with advanced breast cancer and a germline *BRCA* mutation. N Engl J Med.

[236] Huang Y., Chen S., Yao N., Lin S., Zhang J., Xu C. (2024). Molecular mechanism of PARP inhibitor resistance. Oncoscience.

[237] Rose M., Burgess J.T., O'Byrne K., Richard D.J., Bolderson E. (2020). PARP inhibitors: clinical relevance, mechanisms of action and tumor resistance. Front Cell Dev Biol.

[238] Ghosh M., Kang M.S., Katuwal N.B., Hong S.D., Park S.M., Kim S.-G. (2025). SOX5 inhibition overcomes PARP inhibitor resistance in BRCA-Mutated breast and ovarian cancer. Cell Death Dis.

[239] Weigelt B., Comino-Méndez I., de Bruijn I., Tian L., Meisel J.L., García-Murillas I. (2017). Diverse BRCA1 and BRCA2 reversion mutations in circulating cell-free DNA of therapy-resistant breast or ovarian cancer. Clin Cancer Res.

[240] Venetis K., Pepe F., Pescia C., Cursano G., Criscitiello C., Frascarelli C. (2023). ESR1 mutations in HR+/HER2-metastatic breast cancer: enhancing the accuracy of ctDNA testing. Cancer Treat Rev.

[241] Smilkou S., Ntzifa A., Stergiopoulou D., Georgoulias V., Lianidou E. (2024). Multiplex detection of ten ESR1 mutations and AKT1 E17K in breast cancer using digital PCR. J Liq Biopsy.

[242] Guerini-Rocco E., Venetis K., Cursano G., Mane E., Frascarelli C., Pepe F. (2024). Standardized molecular pathology workflow for ctDNA-based ESR1 testing in HR+/HER2- metastatic breast cancer. Crit Rev Oncol Hematol.

[243] Bidard F.-C., Kaklamani V.G., Neven P., Streich G., Montero A.J., Forget F. (2022). Elacestrant (oral selective estrogen receptor degrader) versus standard endocrine therapy for estrogen receptor-positive, human epidermal growth factor receptor 2-Negative advanced breast cancer: results from the randomized phase III EMERALD trial. J Clin Oncol.

[244] Bidard F.-C., Hardy-Bessard A.-C., Dalenc F., Bachelot T., Pierga J.-Y., de la Motte Rouge T. (2022). Switch to fulvestrant and palbociclib versus no switch in advanced breast cancer with rising ESR1 mutation during aromatase inhibitor and palbociclib therapy (PADA-1): a randomised, open-label, multicentre, phase 3 trial. Lancet Oncol.

[245] Bidard F.-C., Mayer E.L., Park Y.H., Janni W., Ma C., Cristofanilli M. (2025). First-line camizestrant for emerging ESR1-Mutated advanced breast cancer. N Engl J Med.

[246] Venetis K., Cursano G., Scafetta R., Giachetti P.P.M.B., Concardi A., De Camilli E. (2025). ESR1 testing on FFPE samples from metastatic lesions in HR +/HER2- breast cancer after progression on CDK4/6 inhibitor therapy. Breast Cancer Res.

[247] Swennenhuis J.F., van Dalum G., Zeune L.L., Terstappen L.W.M.M. (2016). Improving the CellSearch® system. Expert Rev Mol Diagn.

[248] Coumans F., Terstappen L. (2015). Detection and characterization of circulating tumor cells by the CellSearch approach. Methods Mol Biol.

[249] Swennenhuis J.F., van Dalum G., Zeune L.L., Terstappen L.W.M.M. (2016). Improving the CellSearch® system. Expert Rev Mol Diagn.

[250] Coumans F., Terstappen L. (2015). Detection and characterization of circulating tumor cells by the CellSearch approach. Methods Mol Biol.

[251] Jansson S., Bendahl P.-O., Larsson A.-M., Aaltonen K.E., Rydén L. (2016). Prognostic impact of circulating tumor cell apoptosis and clusters in serial blood samples from patients with metastatic breast cancer in a prospective observational cohort. BMC Cancer.

[252] Larsson A.-M., Jansson S., Bendahl P.-O., Levin Tykjaer Jörgensen C., Loman N., Graffman C. (2018). Longitudinal enumeration and cluster evaluation of circulating tumor cells improve prognostication for patients with newly diagnosed metastatic breast cancer in a prospective observational trial. Breast Cancer Res.

[253] Galardi F., De Luca F., Biagioni C., Migliaccio I., Curigliano G., Minisini A.M. (2021). Circulating tumor cells and palbociclib treatment in patients with ER-positive, HER2-negative advanced breast cancer: results from a translational sub-study of the TREnd trial. Breast Cancer Res.

[254] Andree K.C., van Dalum G., Terstappen L.W.M.M. (2016). Challenges in circulating tumor cell detection by the CellSearch system. Mol Oncol.

[255] Wong K.H.K., Tessier S.N., Miyamoto D.T., Miller K.L., Bookstaver L.D., Carey T.R. (2017). Whole blood stabilization for the microfluidic isolation and molecular characterization of circulating tumor cells. Nat Commun.

[256] Ignatiadis M., Sledge G.W., Jeffrey S.S. (2021). Liquid biopsy enters the clinic — implementation issues and future challenges. Nat Rev Clin Oncol.

[257] Trapp E., Janni W., Schindlbeck C., Jückstock J., Andergassen U., de Gregorio A. (2019). Presence of circulating tumor cells in high-risk early breast cancer during Follow-Up and prognosis. JNCI: J Natl Cancer Inst.

[258] Kaldjian E.P., Ramirez A.B., Sun Y., Campton D.E., Werbin J.L., Varshavskaya P. (2018). The RareCyte® platform for next-generation analysis of circulating tumor cells: rarecyte platform CTC analysis. Cytometry.

[259] Dirix L., Buys A., Oeyen S., Peeters D., Liègeois V., Prové A. (2022). Circulating tumor cell detection: a prospective comparison between CellSearch® and RareCyte® platforms in patients with progressive metastatic breast cancer. Breast Cancer Res Treat.

[260] Reduzzi C., Di Cosimo S., Gerratana L., Motta R., Martinetti A., Vingiani A. (2021). Circulating tumor cell clusters are frequently detected in women with early-stage breast cancer. Cancers.

[261] Bharde A., Nadagouda S., Dongare M., Hariramani K., Basavalingegowda M., Haldar S. (2025). ctDNA-based liquid biopsy reveals wider mutational profile with therapy resistance and metastasis susceptibility signatures in early-stage breast cancer patients. J Liq Biopsy.

[262] André F., Ciruelos E., Rubovszky G., Campone M., Loibl S., Rugo H.S. (2019). Alpelisib for *PIK3CA* -Mutated, hormone receptor–positive advanced breast cancer. N Engl J Med.

[263] Davis A.A., Jacob S., Gerratana L., Shah A.N., Wehbe F., Katam N. (2020). Landscape of circulating tumour DNA in metastatic breast cancer. EBioMedicine.

[264] Scheerens H., Malong A., Bassett K., Boyd Z., Gupta V., Harris J. (2017). Current status of companion and complementary diagnostics: strategic considerations for development and launch: options for companion and complementary diagnostics. Clin Trans Sci.

[265] Razavi P., Dickler M.N., Shah P.D., Toy W., Brown D.N., Won H.H. (2020). Alterations in PTEN and ESR1 promote clinical resistance to alpelisib plus aromatase inhibitors. Nat Cancer.

[266] Bidard F.-C., Kaklamani V.G., Neven P., Streich G., Montero A.J., Forget F. (2022). Elacestrant (oral selective estrogen receptor degrader) versus standard endocrine therapy for estrogen receptor-positive, human epidermal growth factor receptor 2-Negative advanced breast cancer: results from the randomized phase III EMERALD trial. J Clin Oncol.

[267] Hoy S.M. (2023). Elacestrant: first approval. Drugs.

[268] André F., Bachelot T., Commo F., Campone M., Arnedos M., Dieras V. (2014). Comparative genomic hybridisation array and DNA sequencing to direct treatment of metastatic breast cancer: a multicentre, prospective trial (SAFIR01/UNICANCER). Lancet Oncol.

[269] Rose Brannon A., Jayakumaran G., Diosdado M., Patel J., Razumova A., Hu Y. (2021). Enhanced specificity of clinical high-sensitivity tumor mutation profiling in cell-free DNA via paired normal sequencing using MSK-ACCESS. Nat Commun.

[270] Bertucci F., Finetti P., Guille A., Adélaïde J., Garnier S., Carbuccia N. (2016). Comparative genomic analysis of primary tumors and metastases in breast cancer. Oncotarget.

[271] Dameri M., Cirmena G., Ravera F., Ferrando L., Cuccarolo P., Stabile M. (2023). Standard operating procedures (SOPs) for non-invasive multiple biomarkers detection in an academic setting: a critical review of the literature for the RENOVATE study protocol. Crit Rev Oncol Hematol.

[272] Fusco N., Jantus-Lewintre E., Serrano M.J., Gandara D., Malapelle U., Rolfo C. (2025). Role of the International Society of Liquid Biopsy (ISLB) in establishing quality control frameworks for clinical integration. Crit Rev Oncol Hematol.

[273] Nuzzo P.V., Rubagotti A., Argellati F., Di Meglio A., Zanardi E., Zinoli L. (2015). Prognostic value of preoperative serum levels of periostin (PN) in early breast cancer (BCa). Int J Mol Sci.

[274] Pistolesi S., Fanelli G.N., Giudice F., Garbini F., Naccarato A.G., Cosio S. (2023). Cervical adenocarcinoma: a still under-investigated malignancy. Anticancer Res.

[275] Coati I., Lotz G., Fanelli G.N., Brignola S., Lanza C., Cappellesso R. (2019). Claudin-18 expression in oesophagogastric adenocarcinomas: a tissue microarray study of 523 molecularly profiled cases. Br J Cancer.

[276] Pasqualetti F., Gonnelli A., Orlandi P., Palladino E., Giannini N., Gadducci G. (2021). Association of XRCC3 rs1799794 polymorphism with survival of glioblastoma multiforme patients treated with combined radio-chemotherapy. Invest N Drugs.

[277] Acosta A.M., Sholl L.M., Fanelli G.N., Gordetsky J.B., Baniak N., Barletta J.A. (2021). Intestinal metaplasia of the urinary tract harbors potentially oncogenic genetic variants. Mod Pathol.

[278] Penney K.L., Tyekucheva S., Rosenthal J., El Fandy H., Carelli R., Borgstein S. (2021). Metabolomics of prostate cancer gleason score in tumor tissue and serum. Mol Cancer Res.

[279] Saraggi D., Galuppini F., Fanelli G.N., Remo A., Urso E.D.L., Bao R.Q. (2018). MiR-21 up-regulation in ampullary adenocarcinoma and its pre-invasive lesions. Pathol Res Pract.

[280] Fassan M., Facchin S., Munari G., Fanelli G.N., Lorenzon G., Savarino E. (2017). Noncoding RNAs as drivers of the phenotypic plasticity of oesophageal mucosa. World J Gastroenterol.

[281] Saraggi D., Galuppini F., Remo A., Urso E.D.L., Bacchin D., Salmaso R. (2017). PD-L1 overexpression in ampulla of vater carcinoma and its pre-invasive lesions. Histopathology.

[282] Davidson S.M., Schmidt D.R., Heyman J.E., O'Brien J.P., Liu A.C., Israelsen W.J. (2022). Pyruvate kinase M1 suppresses development and progression of prostate adenocarcinoma. Cancer Res.

[283] Fassan M., Vianello L., Sacchi D., Fanelli G.N., Munari G., Scarpa M. (2018). Assessment of intratumor immune-microenvironment in colorectal cancers with extranodal extension of nodal metastases. Cancer Cell Int.

[284] Fassan M., Vianello L., Sacchi D., Fanelli G.N., Munari G., Scarpa M. (2019). Correction to: assessment of intratumor immune-microenvironment in colorectal cancers with extranodal extension of nodal metastases. Cancer Cell Int.

[285] Fanelli G.N., Grassini D., Ortenzi V., Pasqualetti F., Montemurro N., Perrini P. (2021). Decipher the glioblastoma microenvironment: the first milestone for new groundbreaking therapeutic strategies. Genes (Basel).

[286] Ma C., Zhou Y., Fanelli G.N., Stopsack K.H., Fiorentino M., Zadra G. (2022). The prostate stromal transcriptome in aggressive and lethal prostate cancer. Mol Cancer Res.

[287] Lasseter K., Nassar A.H., Hamieh L., Berchuck J.E., Nuzzo P.V., Korthauer K. (2020). Plasma cell-free DNA variant analysis compared with methylated DNA analysis in renal cell carcinoma. Genet Med.

[288] Shen S.Y., Singhania R., Fehringer G., Chakravarthy A., Roehrl M.H.A., Chadwick D. (2018). Sensitive tumour detection and classification using plasma cell-free DNA methylomes. Nature.

[289] Pakula H., Omar M., Carelli R., Pederzoli F., Fanelli G.N., Pannellini T. (2024). Distinct mesenchymal cell states mediate prostate cancer progression. Nat Commun.

